# Synthetic circuit for exact adaptation and fold-change detection

**DOI:** 10.1093/nar/gku233

**Published:** 2014-04-11

**Authors:** Jongmin Kim, Ishan Khetarpal, Shaunak Sen, Richard M. Murray

**Affiliations:** 1 Department of Bioengineering, California Institute of Technology, Pasadena, CA 91125, USA; 2 Department of Computer Science, California Institute of Technology, Pasadena, CA 91125, USA; 3 Department of Control and Dynamical Systems, California Institute of Technology, Pasadena, CA 91125, USA

## Abstract

Biological organisms use their sensory systems to detect changes in their environment. The ability of sensory systems to adapt to static inputs allows wide dynamic range as well as sensitivity to input changes including fold-change detection, a response that depends only on fold changes in input, and not on absolute changes. This input scale invariance underlies an important strategy for search that depends solely on the spatial profile of the input. Synthetic efforts to reproduce the architecture and response of cellular circuits provide an important step to foster understanding at the molecular level. We report the bottom-up assembly of biochemical systems that show exact adaptation and fold-change detection. Using a malachite green aptamer as the output, a synthetic transcriptional circuit with the connectivity of an incoherent feed-forward loop motif exhibits pulse generation and exact adaptation. A simple mathematical model was used to assess the amplitude and duration of pulse response as well as the parameter regimes required for fold-change detection. Upon parameter tuning, this synthetic circuit exhibits fold-change detection for four successive rounds of two-fold input changes. The experimental realization of fold-change detection circuit highlights the programmability of transcriptional switches and the ability to obtain predictive dynamical systems in a cell-free environment for technological applications.

## INTRODUCTION

Biological organisms use their sensory systems to respond to changes in their environment. One of the common features found in many sensory systems is exact adaptation in which the output upon change of input to a new constant level gradually returns to a steady level independent of the input (1,2). A well-known example is bacterial chemotaxis in which bacteria in search of nutrient sources are sensitive to a spatial gradient but insensitive to the attractant source levels (3). The basis for exact adaptation and many dynamic regulatory behavior of cells can be found in gene regulatory networks composed of recurring interaction patterns called network motifs (4,5).

**Figure 1.**
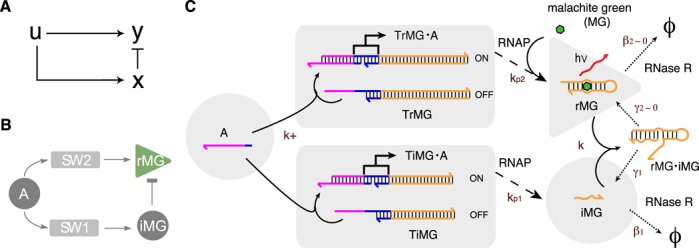
Schematic of synthetic transcriptional adapter. (**A**) A schematic diagram of IFFL motif. (**B**) A schematic diagram of IFFL formed by transcriptional circuit components. The production of two RNA species, malachite green aptamer (rMG) and its inhibitor (iMG), is controlled by the common input A. (**C**) Detailed molecular reactions in the transcriptional circuit. The two templates for rMG and iMG have the same input domain for activator A. The sequence domains are color-coded to indicate identical or complementary sequences: magenta indicates the input domain; dark blue indicates the T7 RNAP promoter; orange indicates the output domain. The hybridization reactions are marked by solid arrows; the production reactions catalyzed by RNAP are marked by dashed arrows; the degradation reactions catalyzed by RNase R are marked by dotted arrows. The binding of malachite green dye to the aptamer rMG is also shown; the fluorescence of MG increases greatly upon binding to the aptamer (this reaction is not modeled because MG is in great excess of the aptamer). The ϕ's indicate the degradation of RNA species to mostly single nucleotides. The reaction rate constants are marked by brown color. We note that two reactions are poorly catalyzed by RNase R by design: the degradation of rMG by RNase R (β_2_ ≃ 0) and the degradation of iMG within rMG·iMG complex by RNase R (γ_2_ ≃ 0).

One of the most common network motifs is the type-I incoherent feed-forward loop (IFFL); the IFFL is a circuit in which a regulator, *u*, activates output target *y* and also activates *x*, a repressor of *y* (Figure 1A). The IFFL is of particular interest because it can generate diverse dynamic features such as a temporal pulse, a band-pass filter and adaptation to gene dosage (6–10). Fold-change detection, where a circuit responds to fold changes in input signals rather than to absolute changes, has been experimentally found in bacterial chemotaxis, wherein it is generated by a specific feedback mechanism (11–14). In theory, fold-change detection can be generated by IFFL within a certain range of parameters, for example, when the intermediate species is a strong repressor of output (15); however, this awaits experimental demonstration (16). Recent studies of cellular responses in Wnt and Erk pathways indicate fold-change detection behaviors in signaling molecular activity (9,17); analogous features were found in *Dictyostelium discoideum* chemotaxis signaling (18,19). Synthetic circuits that demonstrate hallmarks of fold-change detection through IFFL motif provide evidence that this design is sufficient for fold-change detection with biochemically realizable parameters. This helps illustrate that such design may exist in natural biological networks as proposed from previous mathematical analysis and further it could form the basis of exquisite sensing capabilities with biotechnological applications.

DNA-based molecular programs offer a platform for the design of dynamic reactions with arbitrary topologies (20–24). Here, we aim to design a synthetic transcriptional network to implement exact adaptation and fold-change detection using the IFFL circuit motif. As an implementation substrate, we use the ‘genelet’ system, which has been demonstrated to be extremely programmable and versatile (25–28). We start with a simple three-species model and analyze its dynamic features and parameter regimes for fold-change detection. We then rationally design the synthetic adaptation circuit that could be tuned to demonstrate fold-change detection. One of the key requirements in system dynamics was realized by the specific property of RNase R where the degradation rates of RNA molecules can be tuned by the secondary structures at the 3′ end, expanding the repertoire of molecular machinery for synthetic networks. Further, the simple three-dimensional model could explain the experimental results qualitatively when trained using a small subset of experimental results—a step forward from previous more complex models of genelet circuits. This exercise highlights the flexibility and modularity of genelet system and the ease with which we can program biochemical systems in a cell-free environment.

## MATERIALS AND METHODS

### DNA oligonucleotides and enzymes

The sequence of all DNA molecules and expected RNA transcript sequences were chosen to minimize the occurrence of alternative secondary structures checked by the DNA and RNA folding program NUPACK (29). The DNA and RNA sequences used in this study are listed in Supplementary Section S1. (See Figure S1 for sequence domains and predicted secondary structures.) All DNA oligonucleotides and the short RNA signal iMG were purchased from Integrated DNA Technologies (USA). The T7 RNA polymerase (Cellscript, Madison, WI, USA; #C-AS2607), 10× transcription buffer and thermostable inorganic pyrophosphatase (New England Biolabs, Ipswich, MA, USA; #B9012S, #M0296S), NTP and RNase R (Epicentre, Madison, WI, USA; #RN02825, #RNR07250) were purchased. Malachite Green (MG) dye was purchased from Sigma (#M9015). Since pyrophosphatase is involved in regulating the byproduct inorganic pyrophosphate for our transcriptional circuits and is not directly involved in the dynamics, we neglect this enzyme in our models and do not call it an ‘essential enzyme’ for the circuit dynamics. The nominal concentrations of enzyme stocks quoted by the manufacturer were used: 10.5 μM for RNase R and 6 μM for T7 RNA polymerase (RNAP).

### Transcription

DNA templates (T-nt and T-t strands) were annealed with 10% (v/v) 10× transcription buffer from 90°C to 20°C over 1 h at 5 μM concentrations. Transcription reactions were prepared by combining the annealed templates, 7.5 mM each NTP, 24 mM MgCl_2_ (to balance salt concentrations due to increased NTP concentrations), 10% (v/v) 10× transcription buffer and 25 μM MG dye in a test tube. Transcription reactions for spectrofluorometer experiments were prepared as a total volume of 70 μl and enzymes (RNAP, PPase and RNase R) were added and mixed after the baseline fluorescence was recorded for 10 min. DNA activator and inhibitor, A and I, were added from high concentration stocks (3 μM or 30 μM) as inputs. Transcription reactions for gel studies were prepared as a total volume of 50 μl and enzymes (RNAP, PPase, and RNase R) were added and mixed after the control sample (time = 0) was taken. Samples were taken at designated time intervals and the enzyme reactions were stopped by denaturing dye (80% formamide, 10 mM EDTA and 1 mg/ml XCFF). The sample temperature of 33°C was maintained using a four-sample changer with a temperature-controlled water bath for spectrofluorometer experiments and using an incubator for gel experiments.

For the purification of RNA aptamer rMG for fluorescence experiments and for gel controls, the full-length template side strand (the complement of TrMG-nt rather than TrMG-t) was used to prepare a fully duplex DNA template for rMG. The transcription reaction was prepared as a total volume of 60 μl with 0.2 μM fully duplex DNA template. The transcription condition was the same as above except that no A or I were added, 20% (v/v) RNAP was used, and RNase R was omitted. After a 6-h incubation at 37°C, the reaction mixture was treated with 2.5 μl DNase I for 30 min to remove the DNA template, and stopped by the addition of denaturing dye. The reaction mixture was run on 8% denaturing gel, RNA bands were excised and eluted from gel by the crush-and-soak method, ethanol precipitated, and resuspended in water.

### Data acquisition

For spectrofluorometer experiments, excitation and emission for MG fluorescence were at 630 nm and 655 nm, respectively. The fluorescence was recorded every minute using a Fluorolog-3 spectrofluorometer (Jobin Yvon, Edison, NJ, USA) and normalized against the MG fluorescence using a known amount of Malachite Green aptamer (rMG) and MG dye. Denaturing polyacrylamide gels (8% 19:1 acrylamide:bis and 7 M urea in TBE buffer) were allowed to run for 50 min with 10 V/cm at 65°C in TBE buffer (100 mM Tris, 90 mM boric acid and 1 mM EDTA). The 10-base DNA ladder (Invitrogen, Carlsbad, CA, USA; #10821-015) was used as the size marker. The gels were stained with SYBR gold (Molecular Probes, Eugene, OR, USA; #S-11494) and the gel data were quantitated using the Molecular Imager FX (Biorad, Hercules, CA, USA). The total concentrations of RNA product rMG or iMG in the denaturing gel were measured with respect to 1 μM purified rMG or synthesized iMG in control lanes (time = 0).

### Model simulation

The mathematical models were derived from the mechanistic description of DNA/RNA hybridization and enzyme reactions as described in Supplementary Section S2. Simulation of mathematical model and parameter estimation and fittings were implemented in MATLAB. Differential equations were solved using the *ode23s* routine. An initial set of parameters was obtained by kinetic analysis of individual reactions as described in Supplementary Section S3 and later refined by direct fitting of the experimental traces of adapter circuit.

## RESULTS

### Construction of adapter circuit

Using the synthetic transcriptional switch as the regulatory motif and the aptamer for the chromophore MG as the output signal, we construct an IFFL motif (Figure 1B). (Preliminary design and analysis of IFFL circuit was reported in (30).) We designed two transcriptional switches that share common input domains such that they are both activated by a single DNA activator A, serving the role of input *u*. Utilizing the modular switch architecture where the output domain is physically separated from the input domain, an arbitrary output sequence can be encoded irrespective of the input sequence. The two templates transcribe different RNA outputs, rMG and iMG, the MG aptamer and the inhibitor for MG aptamer, serving the role of output *y* and intermediate species *x*, respectively.

Figure 1C illustrates the molecular reactions for the synthetic transcriptional adapter. The OFF state of the synthetic switch consists of a double-stranded (ds) DNA template (‘T’) with a partially single-stranded (ss) and thus incomplete T7 RNAP promoter region (see Supplementary Figure S1 for DNA sequences and detailed diagrams). The two templates encoding rMG and iMG are hereby denoted as TrMG and TiMG. The switches are turned ON by the binding of a ssDNA activator (‘A’) that completes the RNAP promoter region. The resulting template (‘T·A’) has a nicked promoter, but still transcribes well (25). (Although not shown in Figure 1C, an inhibitor strand ‘I’, a perfect complement of A, can initiate binding at the exposed ss domain of A in the ON-state template T·A and strip off A to turn the switch OFF through a toehold-mediated strand displacement reaction (31,32) (Supplementary Figure S1). This reaction involving DNA inhibitor I is implemented only for the downregulation experiment in Figure 5G–I.) RNA outputs will be produced by RNAP from the activated switches using NTP as fuel; the RNA outputs will be degraded by *Escherichia coli* ribonuclease (RNase) R, a single-strand specific RNase. An MG aptamer, rMG, consists of a short RNA sequence whose central loop region serves as the binding pocket for MG. When MG is bound to the aptamer, it becomes highly fluorescent (33). Because the central loop region of the MG aptamer is the binding pocket for MG, disrupting the loop structure abolishes binding of MG to the aptamer. The inhibitor iMG is designed such that it can bind to rMG efficiently by binding to the exposed 5′ end domain of rMG as toehold and initiate branch migration, ultimately disrupting the central loop of rMG upon complex formation (cf. Supplementary Figure S1).

### Mathematical model

To explore the phase space of circuit behavior and to build a theoretical framework to understand the experimental outcome, we constructed a simple mathematical model for the synthetic circuit. The dynamics of this *in vitro* circuit can be described by the five ordinary differential equations for [TiMG·A], [TrMG·A], [iMG], [rMG] and [rMG·iMG] (Supplementary Section S2). (Supplementary Table S1 lists the hybridization and branch migration reactions and the enzyme reactions as shown in Figure 1C.) The system preserves the conservation relations, [T*i*^tot^] = [T*i*] + [T*i*·A], and similarly for [A^tot^], where the superscript tot indicates that all species involving the given strands are being counted. Using these conserved quantities, the remaining variables, [TiMG], [TrMG] and [A], are directly calculated from the concentrations of other species. For fast binding reactions among TiMG, TrMG, and A (large *k* +), the concentration of A reaches zero given [TrMG^tot^] + [TiMG^tot^] > [A^tot^] such that the dynamics of TiMG·A and TrMG·A can be assumed to reach steady-states instantly. Let *u* = [A^tot^], *x* = [iMG], *y* = [rMG] and *z* = [rMG·iMG]. Then a simple three-dimensional model can be derived as follows (Supplementary Section S2):

(1) \begin{eqnarray*} \dot{x}&=& \alpha _1 u-\beta _1 x-kxy+\gamma _1 z, \nonumber \\ \dot{y}&=& \alpha _2 u- kxy, \nonumber \\ \dot{z}&=& kxy- \gamma _1 z, \end{eqnarray*}

where α_1_ and α_2_ are functions of *k*_*p*_'s (RNAP concentration), and the template concentrations $\alpha _1 = k_{p1}\frac{{\rm [TiMG^{tot}]}}{{\rm [TrMG^{tot}]+[TiMG^{tot}]}}$, $\alpha _2 = k_{p2}\frac{{\rm [TrMG^{tot}]}}{{\rm [TrMG^{tot}]+[TiMG^{tot}]}}$; β_1_ and γ_1_ are functions of RNase R concentration and the secondary structure of RNA molecules (due to the binding requirements of RNase R (34), we expect that the degradation of rMG and the degradation of iMG within rMG·iMG complex by RNase R would be negligible, i.e. β_2_ ≃ 0 and γ_2_ ≃ 0 (Supplementary Figures S6 and 7)); *k* is a function of the length of exposed toehold of rMG. Therefore, all of the rate constants are amenable to tuning. The system has a unique equilibrium point if *u* > 0:

(2) \begin{eqnarray*} \bar{x}=\frac{\alpha _1 u}{\beta _1}, \,\,\,\, \bar{y}=\frac{\alpha _2 \beta _1}{\alpha _1 k}, \,\,\,\, \bar{z}=\frac{\alpha _2 u}{\gamma _1}. \end{eqnarray*}

The steady-state value of output *y* in this model is independent of input *u*, showing that the circuit exhibits property of exact adaptation. (When *u* = 0, the steady-states for *x* and *z* are determined to be zero, i.e. $\bar{x}=\bar{z}=0$, but $\bar{y}$ can take any value.)

### Michaelis–Menten enzyme kinetics

One of the limitations in the above mathematical model is the assumed first-order enzyme reactions. A more realistic model is the Michaelis–Menten enzyme reactions, where the available enzyme concentrations depend on the Michaelis constants and substrate concentrations. Using Michaelis–Menten enzyme reactions, the dynamics for the system can be rewritten as follows similar to Equations (1) (Supplementary Section S2):

(3) \begin{eqnarray*} \dot{x}&=& \alpha _1 u f(u) -\beta _1 x g(x,z)-kxy+\gamma _1 z g(x,z), \nonumber \\ \dot{y}&=& \alpha _2 u f(u) - kxy, \nonumber \\ \dot{z}&=& kxy- \gamma _1 z g(x,z), \end{eqnarray*}

where *f*(*u*) and *g*(*x*, *z*) represent the enzyme saturation term for RNAP and RNase R:

(4) \begin{equation*} f(u) =\frac{1}{1+\frac{u}{K_M}} , \,\,\,\, g (x,z) = \frac{1}{1+\frac{x}{K_{M,R,x}}+\frac{z}{K_{M,R,z}}}. \end{equation*}

For the rest of the paper, we will assume that the enzymes are in excess and the substrate concentrations are suitably lower than their respective Michaelis constants such that *f*(*u*) ≈ 1 and *g*(*x*, *z*) ≈ 1, i.e. the dynamics of the system essentially follow Equations (1).

### Non-dimensional equations

To simplify analysis of the design space for this adapter circuit, we rescale variables with respect to the initial input value and the steady-state values,

(5) \begin{equation*} \hat{u}=\frac{u}{u_0}, \,\,\, \hat{x}=\frac{x}{\bar{x}}, \,\,\, \hat{y}=\frac{y}{\bar{y}}, \,\,\, \hat{z}=\frac{z}{\bar{z}}, \end{equation*}

such that all normalized variables have values of 1 at steady-state. (The steady-state for *y* is not determined when the basal input *u*_0_ = 0, and thus, it is not discussed here.) Also, the timescale is normalized by the degradation rate of species *x*: τ = β_1_*t*. Upon change of variables to non-dimensional ones as above (we dropped the $\hat{}$'s with a slight abuse of notations), Equations (1) can be expressed as follows (Supplementary Section S2):

(6) \begin{eqnarray*} \dot{x}&=& u-x-p_1(xy-z), \nonumber \\ \dot{y}&=& p_2(u-xy), \nonumber \\ \dot{z}&=& p_3(xy-z), \end{eqnarray*}

where $p_1=\frac{\alpha _2}{\alpha _1}$, $p_2=\frac{1}{\beta _1}\frac{k \alpha _1 u_0}{\beta _1}$ and $p_3=\frac{\gamma _1}{\beta _1}$. In principle, the two parameters *p*_1_ and *p*_2_ can be arbitrarily tuned by adjusting concentrations of DNA templates, DNA activator and enzymes. On the other hand, *p*_3_ cannot be easily tuned since both β_1_ and γ_1_ are dependent on the concentration of RNase R. (It is possible to differentially adjust β_1_ and γ_1_ by controlling the availability of free 3′ ends of RNA molecules; however, this requires redesign of sequences and may not allow continuous tunability.) The ssRNA iMG as well as the rMG within rMG·iMG complex are designed to have their 3′ ends exposed for longer than 4 nt, and therefore, we expect that the two degradation rates will be similar (cf. Supplementary Table S2).

**Figure 2.**
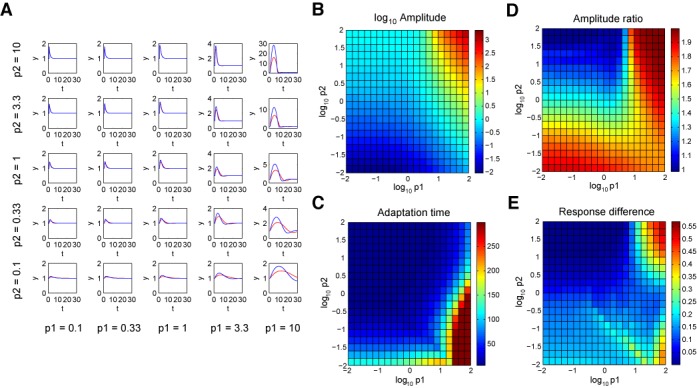
Pulse responses of non-dimensional model for different choice of parameters *p*_1_ and *p*_2_. (**A**) The traces for pulse response of *y*_1_ = *P*(*p*_1_, *p*_2_) (red) and *y*_2_ = *P*(*p*_1_, 2·*p*_2_) (blue), i.e. a trace with twice the baseline input *u*_0_, are shown as *p*_1_ and *p*_2_ are varied. Input *u* changes from 1 to 2 at *t* = 1; all other variables are at steady-states initially, i.e. *x* = *y* = *z* = 1 at *t* = 0. (**B** and **C**) The dependence of pulse amplitude (*A*_1_ = max(*y*_1_) − 1) and adaptation time (*T*_1_ where *y*_1_(*t* > *T*_1_) ∈ [0.99, 1.01]) on *p*_1_ and *p*_2_. (**D** and **E**) The dependence of pulse amplitude ratio (*R* = *A*_2_/*A*_1_) and error between the two traces ($E=\sum _{t=1}^{T_1} |y_1(t)-y_2(t)|/(A_1 \cdot T_1)$) on *p*_1_ and *p*_2_.

With a simplifying assumption that β_1_ = γ_1_, i.e. *p*_3_ = 1, we can map out the phase space of adapter circuit as functions of *p*_1_ and *p*_2_ (Figure 2). Upon change of *u* from 1 to 2 at *t* = 1, example traces for the response of *y* as the values of *p*_1_ and *p*_2_ change are shown in Figure 2A (*y*_1_ = *P*(*p*_1_, *p*_2_); red curves). The amplitude of pulse increases as both *p*_1_ and *p*_2_ become large; the adaptation time decreases as *p*_1_ becomes small but *p*_2_ becomes large (Figure 2B and C). Further, we can map out whether the adapter circuit can show fold-change detection as functions of *p*_1_ and *p*_2_. To satisfy fold-change detection criteria, the circuit should produce the same output responses for the same fold change of inputs, e.g. an input change from 1 to 2 versus an input change from 2 to 4. These two cases are identical in terms of change in *u* (normalized input fold) from 1 to 2, however, their starting input *u*_0_ differ by a factor of 2—this difference in *u*_0_ is captured by the difference in *p*_2_ in the non-dimensional model. Thus, we need to compare the two traces *y*_1_ = *P*(*p*_1_, *p*_2_) and *y*_2_ = *P*(*p*_1_, 2·*p*_2_) as functions of *p*_1_ and *p*_2_: example traces for the response of *y*_1_ (red) and *y*_2_ (blue) are shown in Figure 2A. The ratio of amplitudes for the two traces is close to 1 as *p*_1_ becomes small and *p*_2_ becomes large, indicating fold-change detection; similarly, the error between the two traces decreases as *p*_1_ becomes small and *p*_2_ becomes large (Figure 2D and E).

### Fold-change detection

Given small *p*_1_ and large *p*_2_, Equations (6) can be approximated by

(7) \begin{equation*} \dot{x}=u-x, \,\,\,\, y=u/x. \end{equation*}

(Note that large *p*_3_ helps this approximation by accelerating dynamics of *z*; however, small *p*_3_ can make this approximation inaccurate.) Upon change of variables to *F* = *u*/*u*_0_, *X* = *x*/*u*_0_, the equation above can be rewritten as

(8) \begin{equation*} \dot{X}=F-X, \,\,\,\, y=F/X. \end{equation*}

Therefore, the dynamics of the system depends solely on the fold change of the input *u* (15). We note that the non-dimensional model (Equations (6)) shows approximate fold-change detection for a wide range of parameter choices (Figure 2).

### Characterization of elementary reactions

To verify the underlying assumptions used to construct the model for functional transcriptional adapter, we performed basic characterization of elementary reactions. In addition to gaining mechanistic understanding of the circuit, we can use the estimates for reaction rates thus obtained as parameter values for the mathematical model of transcriptional adapter.

We observed that the bleaching of free MG dye in the transcription reaction buffer is not negligible for long experiments (Supplementary Figure S2). The bleaching of MG dye is reported to follow a pseudo-first-order kinetics (35); thus, an exponential decay curve was used for background correction for all fluorescence trajectories. Known concentrations of purified MG aptamer were used to convert the fluorescence reading to aptamer concentrations after background correction.

**Figure 3.**
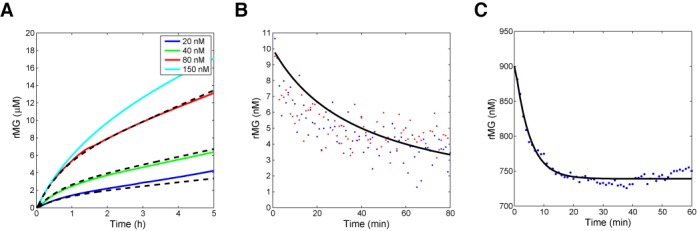
Characterization of elementary reactions. (**A**) Determination of transcription rates for rMG (α_2_). Fluorescence of rMG transcripts was converted to rMG concentrations using offline measurements of fluorescence signals from purified rMG as standards. The experimental conditions were as follows: [TrMG^tot^] = 50 nM for low inputs (20 and 40 nM), [TrMG^tot^] = 150 nM for high inputs (80 and 150 nM), and [RNAP] = 171 nM. The fit results using burst phase model for activator concentrations ranging from 20 to 80 nM are shown as black dashed lines. (**B**) Determination of binding rates for rMG and iMG (*k*). The hybridization reaction of rMG and iMG was characterized by adding 10 nM of iMG to 10 nM of rMG. The data points for duplicate reactions are plotted as red and blue dots. The signal was noisy due to the low concentration of rMG used. The black line is the fit ([rMG] = 1/(*kt* + 0.1)). (**C**) Determination of degradation rates for rMG·iMG complex (β_1_ and γ_1_). The degradation reaction of rMG·iMG complex was characterized by adding 75 nM of RNase R to 100 nM of iMG and 1 μM rMG. The degradation of rMG stopped in the middle presumably because all the iMG molecules were exhausted. The black line is the fit using a simple model for hybridization and degradation. See Supplementary Section S3 for details.

First, the transcription reactions for the two templates, TrMG and TiMG, are characterized through fluorescence measurements. Upon the addition of different concentrations of activators, rMG is produced from TrMG·A, increasing the fluorescence signal (Figure 3A), while iMG is produced from TiMG·A, decreasing the fluorescence of rMG by opening up the binding pocket for MG dye (Supplementary Figure S4). The steady-state transcription rates for rMG and iMG showed linear dependence on the activator concentration used (Supplementary Figures S3 and S4). Interestingly, the transcription rate of rMG was apparently not constant for the initial part of transcription reaction reminiscent of ‘burst phase’ in enzyme reaction (36). Thus, we use the following equation to fit the transcription rate for rMG:

(9) \begin{equation*} k_{p2,{\rm burst}}=k_{p2}(1+B e^{-t/\tau _b}), \end{equation*}

where *B* is the magnitude of increased transcription efficiency during the initial burst phase and τ_*b*_ is the time constant for duration of burst phase. This burst equation can be used to phenomenologically fit the strong first pulse response of adapter circuit.

**Figure 4.**
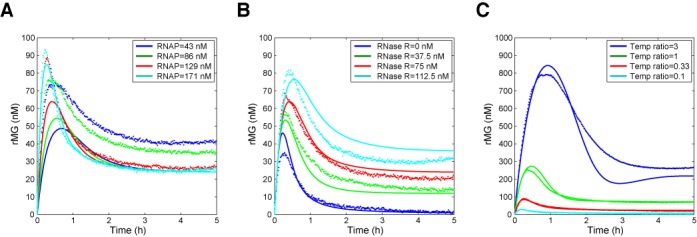
Experimental and simulation results for circuit responses for different concentrations of enzymes and template ratios. The pulse response of adapter circuit was characterized for (**A**) RNAP variation, (**B**) RNase R variation, and (**C**) template ratio [TrMG^tot^]/[TiMG^tot^] variation with the total concentration of templates fixed at 200 nM. The template concentrations were [TrMG^tot^] = 50 nM and [TiMG^tot^] = 150 nM for (A) and (B); RNAP concentration was 171 nM for (B) and (C); RNase R concentration was 75 nM for (A) and (C). The simple model (Equations (1)) was used for simulation with the ‘optimized’ parameters listed in Supplementary Table S2. The simulation results captured the features of experimental results (cf. Table 1). The experimental data are plotted as dots and the simulation results are plotted as lines.

Next, the binding reaction for the two RNA signals, rMG and iMG, is characterized through fluorescence monitoring (Figure 3B and Supplementary Figure S5). The observed binding rate monitored as fluorescence decay was consistent with typical toehold-mediated branch migration reactions (31,32).

Finally, we characterized the degradation of RNA signals catalyzed by RNase R. Due to the binding requirements of RNase R, RNA sequences with less than 4 nt of ss regions exposed at the 3′ end would be degraded poorly by RNase R (34). To minimize the degradation rate of rMG by RNase R (β_2_), we placed a single unpaired base at the 3′ end of rMG (Figure 1C and Supplementary Figure S1). The degradation rate of rMG by RNase R was monitored by fluorescence and gel (Supplementary Figure S6); the degradation of rMG alone by RNase R was negligible (β_2_ ≃ 0). On the other hand, the other RNA signal iMG is designed to have no significant secondary structure, presumably providing a long ss region at its 3′ end (Figure 1C and Supplementary Figure S1). The degradation of iMG alone by RNase R was fast from gel analysis (data not shown). Then, the degradation pathway for the rMG·iMG complex was investigated. Gel analysis of preformed rMG·iMG complex incubated with RNase R showed that both rMG and iMG were degraded quickly (Supplementary Figure S7). Thus, we conclude that the degradation of iMG within rMG·iMG complex is very slow (γ_2_ ≃ 0). (Because stoichiometric amount of rMG and iMG were used for gel analysis, if iMG were to be degraded first (γ_2_ > γ_1_), rMG would be left over which cannot be easily degraded (β_2_ ≃ 0).) Quantitative estimates for parameter values were obtained from the spectrofluorometer experiment where a substoichiometric amount of iMG was used with respect to rMG (Figure 3C and Supplementary Figure S7). The iMG molecules were able to catalyze degradation of rMG beyond its stoichiometric ratio, suggesting that the iMG molecule released upon degradation of rMG within the rMG·iMG complex can bind to free rMG molecules such that it catalyzes the degradation of multiple rMG molecules. Therefore, the design strategy that limits exposure of 3′ end of iMG upon rMG·iMG complex formation while releasing the hidden 3′ end of rMG results in fast degradation of rMG within rMG·iMG complex as desired (γ_1_ ≫ 0 and γ_2_ ≃ 0). The parameter estimates are listed in Supplementary Table S2.

Together, the characterization of elementary reactions verified the following properties: first, the transcription rates are linear with respect to the input activator concentrations; second, the binding reaction of rMG and iMG is fast and effectively disrupts the binding pocket for MG dye; third, the degradation rate of iMG is fast while the degradation rate of rMG is slow; fourth, the degradation of rMG·iMG presumably results in iMG, which is subsequently degraded or binds to free rMG molecule. All of these properties are consistent with the simple mathematical model, indicating that the simple model can potentially be used to explain most of the circuit behaviors quantitatively.

### Characterization of pulse responses

Here, all the components required for a functional adapter are put together and experimentally tested at the system level. We use the simple mathematical model and compare the simulation results with the performance of transcriptional adapter.

First, we characterize how the circuit response depends on the concentration of RNAP. Upon the addition of 20 nM input A at time 0, the rMG signal initially increases, peaks, and then decreases to a stable steady-state for all RNAP concentrations tested (Figure 4A). From the simple mathematical model (Equations (1)), if the increase of RNAP increased both transcription rate constants *k*_*p*1_ and *k*_*p*2_ to the same extent, we expect the steady-state rMG level to be unchanged. The steady-state rMG level increased for low RNAP in the experiment, indicating that the assumed linear dependence of transcription rate constants on RNAP concentration may not be accurate for low RNAP concentrations. High RNAP concentrations reduced the response time of rMG in the experiment, consistent with simple model predictions where increased RNAP increases iMG concentration, and thus increases the ‘effective’ degradation rate constant for rMG (Table 1). We chose to use high RNAP concentration (171 nM) throughout the experiments.

**Table 1.** Expected and observed response characteristics of rMG

	Steady-state	Response time	Parameter values
[RNAP] ↑	sim − versus exp ↓	sim ↓ versus exp ↓	α_1_↑, α_2_↑
[RNase R] ↑	sim ↑ versus exp ↑	sim ↑ versus exp ↑	β_1_↑, γ_1_↑
[TrMG^tot^] ↑, [TiMG^tot^] ↓	sim ↑ versus exp ↑	sim ↑ versus exp ↑	α_1_↓, α_2_↑

Note that $\alpha _1=\frac{k_{p1}[{\rm TiMG^{tot}}]}{[{\rm TrMG^{tot}}]+[{\rm TiMG^{tot}}]}$ , $\alpha _2=\frac{k_{p2}[{\rm TrMG^{tot}}]}{[{\rm TrMG^{tot}}]+[{\rm TiMG^{tot}}]}$, and *u* = [A^tot^] such that $[{\rm {rMG}}_{{{\rm ss}}}] = \frac{\alpha _2 \beta _1}{\alpha _1 k} = \frac{k_{p2} \beta _1}{k_{p1} k}\frac{[{\rm TrMG^{tot}}]}{[{\rm TiMG^{tot}}]}$, $[{\rm iMG}_{{\rm ss}}] = \frac{\alpha _1 u}{\beta _1} = \frac{k_{p1}[{\rm TiMG^{tot}}]}{[{\rm TrMG^{tot}}]+[{\rm TiMG^{tot}}]}\frac{[{\rm A^{tot}}]}{\beta _1}$.

We also assume that *k*_*p*1_, *k*_*p*2_∝[RNAP] and β_1_, γ_1_∝[RNase R] here.

Next, we characterize how the circuit response depends on the concentration of RNase R. Upon the addition of 10 nM input A, pulse generation was observed for all RNase R concentrations tested (Figure 4B). The steady-states of rMG concentrations were almost proportional to the degradation rate by RNase R (β_1_); high degradation rate by RNase R leads to smaller iMG concentration slowing down the response time of rMG as expected (Table 1). We determined that 75 nM RNase R concentration led to an adequate steady-state output level as well as a reasonably fast response time. Thus, we decided to use 75 nM of RNase R throughout the experiments.

We further explore whether the adaptation behavior is robust to variations in the template ratios. The template ratios ([TrMG^tot^]/[TiMG^tot^]) were varied from 0.1 to 3 while keeping the total concentration of templates fixed at 200 nM. Upon the addition of 20 nM input A at time 0, the rMG signals showed pulse generation and stabilization to steady-states (Figure 4C). The steady-state of rMG concentrations were almost proportional to the template ratio as expected; this is shown clearly by the fluorescence traces normalized according to the expected steady-state rMG values (Supplementary Figure S8). The response time of rMG increases as the template ratio increases consistent with the simple model prediction (Table 1).

With the ‘default’ set of parameters obtained from characterization of elementary reactions (Supplementary Table S2), the dynamic behavior predicted by the simple mathematical model (Equations (1)) showed qualitatively correct trends in terms of steady-state values and timescale of adaptation for rMG signals (Supplementary Figure S9). It is not surprising that we do not obtain a quantitative agreement considering the approximations made both in building the model and in measuring its kinetic parameters. After a simple fitting process to minimize least-squared error, the model described the experimental data set much more accurately over the range of enzyme and template concentrations (Figure 4). Interestingly, the simulation results of a more detailed model where both switch templates are modeled as distinct states (Supplementary Figure S9) were indistinguishable from the simulation results of the simple model because the fitted *k*_+_ was fast as we have assumed in deriving the simple model.

Concerning the parameters' values, we note that it was possible to obtain this good fit starting from the initial ‘default’ parameter set (Supplementary Table S2). The largest adjustment was required for degradation rates, β_1_ and γ_1_, where these rate constants were adjusted to be much lower than the values estimated from elementary reactions. This discrepancy may be in part due to the incomplete transcription products (abortive transcripts) generated by RNAP (37). These abortive transcripts (typically 8–13 bases long 5′ end fragments of rMG and iMG) are not likely to participate in the designed reaction pathways; however, they are good substrates for RNase R since these products would not have strong secondary structures, and therefore they will ‘effectively’ decrease the degradation rates for the correct transcription products. Indeed, gel analysis of transcription products revealed several incomplete transcripts and potential extension products (Supplementary Figure S10), helping explain the discrepancy as compared with elementary reaction characterization where purified rMG and iMG were used as substrates. Another possibility includes non-specific binding among enzymes and DNA/RNA species. The binding rate constant *k* also decreased slightly after optimization; this may in part be due to interference from incomplete transcripts and/or incomplete degradation products as well as the potential non-specific binding of enzymes. The parameters for transcription reactions, *k*_*p*1_, *k*_*p*2_, *B* and τ_*b*_, were given a relatively narrow range to explore because we expect that the simple characterization steps would have resulted in reasonable parameter estimates (the production of incomplete transcripts for rMG does not contribute to fluorescence and similarly incomplete iMG molecules will not disrupt the binding pocket of rMG, although both incomplete products may constitute a significant portion of transcripts); none of them deviated a lot from the initial parameter estimates. To match the initial parts of pulses, the ‘burst phase’ model of RNAP where the first few turnovers are faster than the steady-state outputs played an important role (cf. Figure 3A).

Previous studies indicated that the enzyme activity varies by a few fold depending on the batch of enzyme used and that such variability can affect functionality of synthetic transcription circuits in many aspects (25–28). Therefore, we tested whether the circuit performance is repeatable and robust to change of enzyme activities originating from batch-to-batch variability. The template ratio variation experiments—the same as outlined in Figure 4C—were performed in triplicate with a new batch of RNAP and RNase R (Supplementary Figure S11). While not quantitatively the same as Figure 4C, the predicted trends for the pulse amplitude and adaptation time were correct for a wide range of template ratios. The average standard deviations ranged from 12 to 16% of the final steady-state in each experiment, indicating that the adaptation behavior is reproducible. (Note that the new batch of enzymes were used only for the results presented in Supplementary Figures S11 and S13.)

### Response to multiple input perturbations

**Figure 5.**
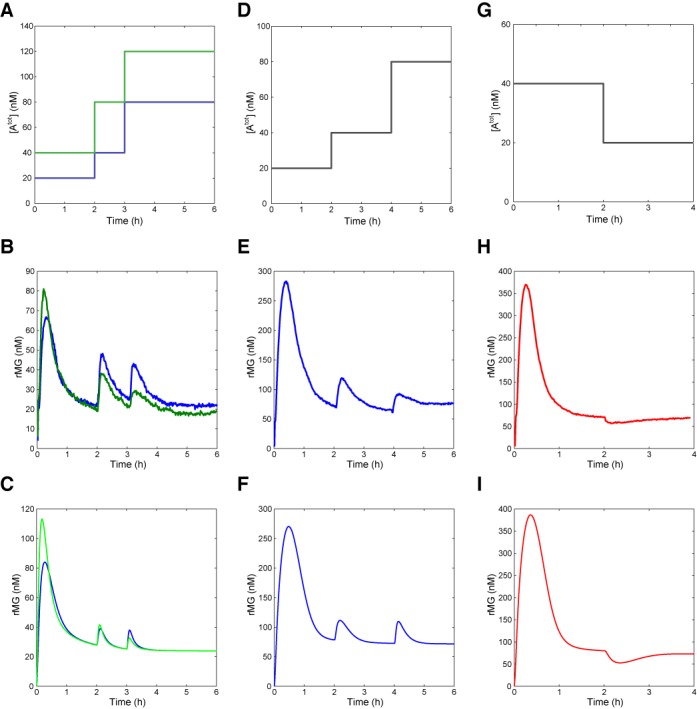
Circuit responses for multiple inputs. (**A–C**) After the initial pulse response settled down, two input perturbations were induced at 2 h and 3 h. The template concentrations were [TrMG^tot^] = 50 nM and [TiMG^tot^] = 150 nM. (**D–F**) Two input perturbations were induced at 2 h and 4 h with [TrMG^tot^] = [TiMG^tot^] = 100 nM. (**G–I**) Circuit response to a negative input (inhibitor I) was tested with [TrMG^tot^] = [TiMG^tot^] = 100 nM. Top row: input profiles; middle row: experimental results; bottom row: simulations.

We then explore whether the adaptation response is robust to multiple input perturbations. The addition of 20 nM input A at the beginning was followed by the addition of 20 nM input A at 2 h and 40 nM at 3 h (Figure 5A, blue). Note that the total amount of input increased from 20 nM to 40 nM and then to 80 nM: observing a sequence of 2-fold increases. Interestingly, the addition of the last input elicited a similar response in rMG signal despite being at higher concentration than the second input (Figure 5B, blue). This suggests that the system can show an approximate fold-change detection. In another experimental trace, the addition of 40 nM input A at the beginning was followed by the addition of 40 nM input A at 2 h and 40 nM at 3 h (Figure 5A, green). In this case, the addition of the last input elicited a smaller response than the second input (Figure 5B, green). The simulation result showed that the second input perturbations in both cases elicited similar magnitude of responses despite the 2-fold differences in concentrations (Figure 5C). This indicates that the fold difference of the current input as compared to the previous input rather than the concentration of the current input itself is an important factor for circuit dynamics.

Multiple input perturbations were used to test the robustness of adaptation when the two template concentrations were equal. The addition of 20 nM input A at the beginning was followed by the addition of 20 nM input A at 2 h and 40 nM at 4 h (Figure 5D). Interestingly, the addition of the last input elicited a smaller response in rMG signal despite being at higher concentration than the second input (Figure 5E). (In fact, the input sequence is the same as that of blue curve in Figure 5A.) This indicates that an increased template ratio [TrMG^tot^]/[TiMG^tot^] moved the system away from the parameter regime where an approximate fold-change detection can be observed. On the other hand, the simulation result still showed an approximate fold-change detection for the final input perturbation (Figure 5F).

The inhibition mechanism of DNA templates by DNA inhibitor I (a perfect complement of input A) could be used to implement negative inputs. The addition of 40 nM input A at the beginning was followed by the addition of 20 nM inhibitor I at 2 h (Figure 5G). The expected downswing in the rMG signal was observed, which subsequently returned to the previous steady-state level by 4 h (Figure 5H). The simulation result showed reasonable agreement with the experimental trace (Figure 5I).

### Fold-change detection

**Figure 6.**
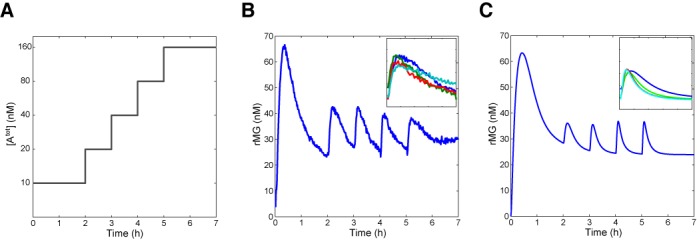
Circuit response follows fold change of inputs. (**A**) After the initial transient was settled, multiple input perturbations were induced every hour; the total concentration of inputs were increased by 2-fold every time: [A^tot^] = 10 nM at time 0, 20 nM at 2 h, 40 nM at 3 h, 80 nM at 4 h, and 160 nM at 5 h (shown as log-scale in y-axis). (**B**) The fluorescence signal of rMG showed approximately identical pulse responses upon repeated 2-fold input changes. The segments of timecourse are rearranged with respect to time after input perturbations indicating an approximate fold-change detection (inset). (**C**) The corresponding simulation results are shown. The template concentrations were [TrMG^tot^] = 50 nM and [TiMG^tot^] = 150 nM.

Finally, we tested whether the experimental results bear out the fold-change detection property for appropriate parameter choices as predicted by model. In principle, the two parameters *p*_1_ and *p*_2_ in the non-dimensional model (Equations (6)) can be arbitrarily tuned, allowing easy exploration of circuit dynamics: $p_1=\frac{\alpha _2}{\alpha _1}$ and $p_2=\frac{1}{\beta _1}\frac{k \alpha _1 u_0}{\beta _1}$. (Practically, however, too small *p*_1_ can be problematic due to detection limit of the instrument and the significant bleaching at very low signal level.) Using the optimized parameter values (Supplementary Table S2), the experimental condition corresponded to *p*_1_ = 0.19, *p*_3_ = 0.61, and an initial value of *p*_2_ = 0.93 after the first injection of 10 nM input A. (Note that the initial value of *p*_2_ is small for fold-change detection; however, the value of *p*_2_ increases as more input A is injected to the circuit, approaching the parameter regime for fold-change detection.) After the initial transient induced by 10 nM input A settled down at 2 h, the following sequence of additional input A was used every hour: 10, 20, 40 and 80 nM. Note that the total concentration of input A changed by a factor of two after each injection of input: [A^tot^] = 10 nM at time 0, 20 nM at 2 h, 40 nM at 3 h, 80 nM at 4 h and 160 nM at 5 h (Figure 6A). The experimental results show that the circuit response follows an approximate fold-change detection in good agreement with model predictions (Figure 6B); this is shown clearly by the overlap of fluorescence traces for the four separate input injections (Figure 6B, inset). The simulation results showed an approximate fold-change detection as well (Figure 6C), in which the dynamics of pulse response converged as more input is introduced to the system (Figure 6C, inset). Similarly, an experiment with all input concentrations halved showed an approximate fold-change detection (Supplementary Figure S12). Further, experiments with a new set of enzyme batches showed an approximate fold-change detection with small variability for triplicate measurements, indicating that the fold-change detection is reproducible (Supplementary Figure S13).

## DISCUSSION

IFFLs represent important biomolecular circuit elements capable of a rich set of dynamic behavior including pulsing, adaptation and fold-change detection (16). Using previously developed genelet toolbox (25), we present a systematic construction of such a circuit, characterizing both the parameter values as well as the circuit behavior with high precision fluorescence measurements. First, we present and systematically characterize a mathematical model for the realization of an IFFL circuit that can exhibit pulses, adaptation and fold-change detection. This aids the design of a biomolecular realization through insight into parametric regimes of desired behavior. Second, we characterize the elementary reactions to verify underlying model assumptions and establish the feasibility of observing required dynamics. Third, we measure each of the three key properties of the IFFL—pulses, adaptation and fold-change detection—which can be shown to match the model predictions qualitatively. These results present a rational and systematic design strategy of a dynamically rich synthetic circuit.

Although digital abstraction has been often employed for building and understanding synthetic biological circuits (38), analog functions are pervasive in natural biological systems (39). Recently, by exploiting analog building-block functions already present in cells, synthetic circuits with logarithmically linear sensing capabilities were implemented (40). The ability to detect fold change of signals is closely related to logarithmic sensing, suggesting a potential for analog computations using IFFL motif.

For the general application of such circuit architecture, it is important to understand the working parameter regime—when and where the simplifying assumptions and composability with other elements may fail. While the parameters can be tuned to achieve approximate fold-change detection, we need to keep in mind that the first-order production and degradation terms are valid only when the enzymes are in excess and/or the substrate concentrations are suitably low. The saturation on the production enzyme does not impact adaptation property per se, but the saturation on the degradation enzyme precludes an exact adaptation behavior. This limits the extension of the current circuit architecture in case high concentrations of downstream RNA signals targeted by RNase R are desired. While RNase R has been utilized in parallel with RNase H for synthetic circuits (27), the degradation products generated by RNase H could induce further load on the limited capacity of RNase R. It remains to be seen how the variety of natural RNases with diverse substrate specificities (41) can be utilized for synthetic circuit functionality.

Here, we focus on the use of RNase R as the degradation machinery of choice. An interesting feature of RNase R distinct from other RNases is that it can by itself degrade RNAs with extensive secondary structure provided that more than 4 nt of ss 3′ overhang is present, while duplex RNAs with no overhang binds to RNase R with a dissociation constant greater than 5 μM (34). Therefore, employing different secondary structures for RNA substrates at their 3′ ends offers an opportunity to tune degradation rates by RNase R by utilizing native RNA structures and designed extension of 3′ ends. Further, the degradation rates of target species can be controlled by another species that alters the availability of 3′ ends as exemplified in this work. The ability to control degradation rates allows more sophisticated control of dynamic behavior and could also be used to minimize maintenance energy costs—e.g., by ramping up the production rate only for the duration of switching states from OFF to ON and ramping up the degradation rate only for switching from ON to OFF—much as in complementary metal–oxide–semiconductor (CMOS) technology for electrical circuits with low static power consumption.

Nucleic acids are extremely versatile chemical building blocks with structural, mechanical, recognition and catalytic capabilities. DNA origami (42,43), DNA gels (44,45), aptamers (46), ribozymes (47) and circuits (48–51) offer a wide selection of basic units for design and construction of complex molecular behavior. Further, genelets have been used to control nucleic-acid-based molecular structure and aptamer in a straightforward manner (28), opening up a rich possibility for spatiotemporal control of nucleic-acid-based molecular programs and nanostructures embedded in artificial cells (52).

## SUPPLEMENTARY DATA

Supplementary Data are available at NAR Online, including Supplementary References [1–13].

SUPPLEMENTARY DATA
