# Supplementary material for: Synthetic circuit for exact adaptation and fold-change detection
Source: Nucleic Acids Res. 2014 Apr 11;42(9):6078–89. doi: 10.1093/nar/gku233 (PMC4027175; doi:10.1093/nar/gku233)
Supplement: SUPPLEMENTARY DATA [file supp_42_9_6078__index.html]

Synthetic circuit for exact adaptation and fold-change detection — Synthetic circuit for exact adaptation and fold-change detection — SUPPLEMENTARY DATA 

# Synthetic circuit for exact adaptation and fold-change detection

## SUPPLEMENTARY DATA

**Files in this Data Supplement:**

- Supplementary Data
