# Supplementary material for: Synthetic circuit for exact adaptation and fold-change detection
Source: Nucleic Acids Res. 2014 Apr 11;42(9):6078–89. doi: 10.1093/nar/gku233 (PMC4027175; doi:10.1093/nar/gku233)
Supplement: SUPPLEMENTARY DATA [file supp_gku233_nar-02987-n-2013-File001.pdf]

Synthetic circuit for exact adaptation and fold-change detection
Supporting Information

Jongmin Kim[1,*], Ishan Khetarpal[2], Shaunak Sen[3,‡], Richard M. Murray[1,3]
Departments of **1** Bioengineering, **2** Computer Science, and **3** Control and Dynamical Systems,
California Institute of Technology, Pasadena, CA 91125, USA
‡ Present address: Department of Electrical Engineering, Indian Institute of Technology Delhi,
New Delhi, India
∗ Correspondence: jongmin@dna.caltech.edu

1. Methods

The sequence of DNA oligonucleotides and RNA outputs are listed as follows. See Figure S1 for sequence domains and predicted secondary structures.

DNA sequences

TrMG-nt (84mer), 5′-CTAATGAACTACTACTACACACTAATACGACTCACTATAGGACAAGCATCCCGACTGGCGAGAGCCAGGTAAC-GAATGGATGCG-3′.
TrMG-t (57mer), 5′-CGCATCCATTCGTTACCTGGCTCTCGCCAGTCGGGATGCTTGTCCTATAGTGAGTCG-3′.
TiMG-nt (56mer), 5′-CTAATGAACTACTACTACACACTAATACGACTCACTATAGGTCGGGATGCTTGTCT-3′.
TiMG-t (29mer), 5′-AGACAAGCATCCCGACCTATAGTGAGTCG-3′.
A (35mer), 5′-TATTAGTGTGTAGTAGTAGTTCATTAGTGTCGTTC-3′.
I (35mer), 5′-GAACGACACTAATGAACTACTACTACACACTAATA-3′.

RNA output sequences

rMG (45mer), 5′-GGACAAGCAUCCCGACUGGCGAGAGCCAGGUAACGAAUGGAUGCG-3′.
iMG (17mer), 5′-GGUCGGGAUGCUUGUCU-3′.

2. Mathematical Model

To explore the phase space of circuit behavior and to build theoretical framework to understand experimental outcome, we constructed a simple mathematical model for the synthetic circuit. Table S1 lists the hybridization and branch migration reactions and the enzyme reactions as shown in Figure 1C. Here, we do not consider side reactions or incomplete transcription and degradation products. Also for the simple model presented here, the enzyme reactions are treated as approximately first-order reactions. The dynamics of this *in vitro* circuit can be described by the following five ordinary differential equations:

$$\begin{aligned}
\frac{d}{dt}[\mathrm{TiMG}\cdot\mathrm{A}] &= k_+[\mathrm{TiMG}][\mathrm{A}],\\
\frac{d}{dt}[\mathrm{TrMG}\cdot\mathrm{A}] &= k_+[\mathrm{TrMG}][\mathrm{A}],\\
\frac{d}{dt}[\mathrm{iMG}] &= k_{p1}[\mathrm{TiMG}\cdot\mathrm{A}] - \beta_1[\mathrm{iMG}] - k[\mathrm{iMG}][\mathrm{rMG}] + \gamma_1[\mathrm{rMG}\cdot\mathrm{iMG}],\\
\frac{d}{dt}[\mathrm{rMG}] &= k_{p2}[\mathrm{TrMG}\cdot\mathrm{A}] - \beta_2[\mathrm{rMG}] - k[\mathrm{iMG}][\mathrm{rMG}] + \gamma_2[\mathrm{rMG}\cdot\mathrm{iMG}],\\
\frac{d}{dt}[\mathrm{rMG}\cdot\mathrm{iMG}] &= k[\mathrm{rMG}][\mathrm{iMG}] - \gamma_1[\mathrm{rMG}\cdot\mathrm{iMG}] - \gamma_2[\mathrm{rMG}\cdot\mathrm{iMG}]. \qquad (1)
\end{aligned}$$

The system preserves the conservation relations, $[\mathrm{T}i^{\mathrm{tot}}] = [\mathrm{T}i] + [\mathrm{T}i\cdot\mathrm{A}]$, and similarly for $[\mathrm{A}^{\mathrm{tot}}]$, where the superscript tot indicates that all species involving the given strands are being counted. Using these conserved quantities, the remaining variables, [TiMG], [TrMG] and [A], are directly calculated from the concentrations of other species. Note that the binding reactions of templates and activator A are simple binding reactions where the concentration of A decreases to ≈ 0 over time if $[\mathrm{TrMG}^{\mathrm{tot}}] + [\mathrm{TiMG}^{\mathrm{tot}}] > [\mathrm{A}^{\mathrm{tot}}]$; for fast binding reactions (large $k+$), the dynamics of A can be treated as essentially at steady-states. Let $u = [\mathrm{A}^{\mathrm{tot}}]$, $x = [\mathrm{iMG}]$, $y = [\mathrm{rMG}]$, and $z = [\mathrm{rMG}\cdot\mathrm{iMG}]$. Then the eqs. (1) can be rewritten as follows:

$$\begin{aligned}
\dot{x} &= \alpha_1 u - \beta_1 x - kxy + \gamma_1 z,\\
\dot{y} &= \alpha_2 u - \beta_2 y - kxy + \gamma_2 z,\\
\dot{z} &= kxy - \gamma_1 z - \gamma_2 z, \qquad (2)
\end{aligned}$$

where α_1 and α_2 are functions of k_p's (RNAP concentration), $[\mathrm{TrMG}^{\mathrm{tot}}]$, and $[\mathrm{TiMG}^{\mathrm{tot}}]$, β_1, β_2, γ_1, and γ_2 are functions of RNase R concentration and the secondary structure of RNA molecules, and k is a function of the length of exposed toehold of rMG. Therefore, all of the rate constants are amenable to tuning.

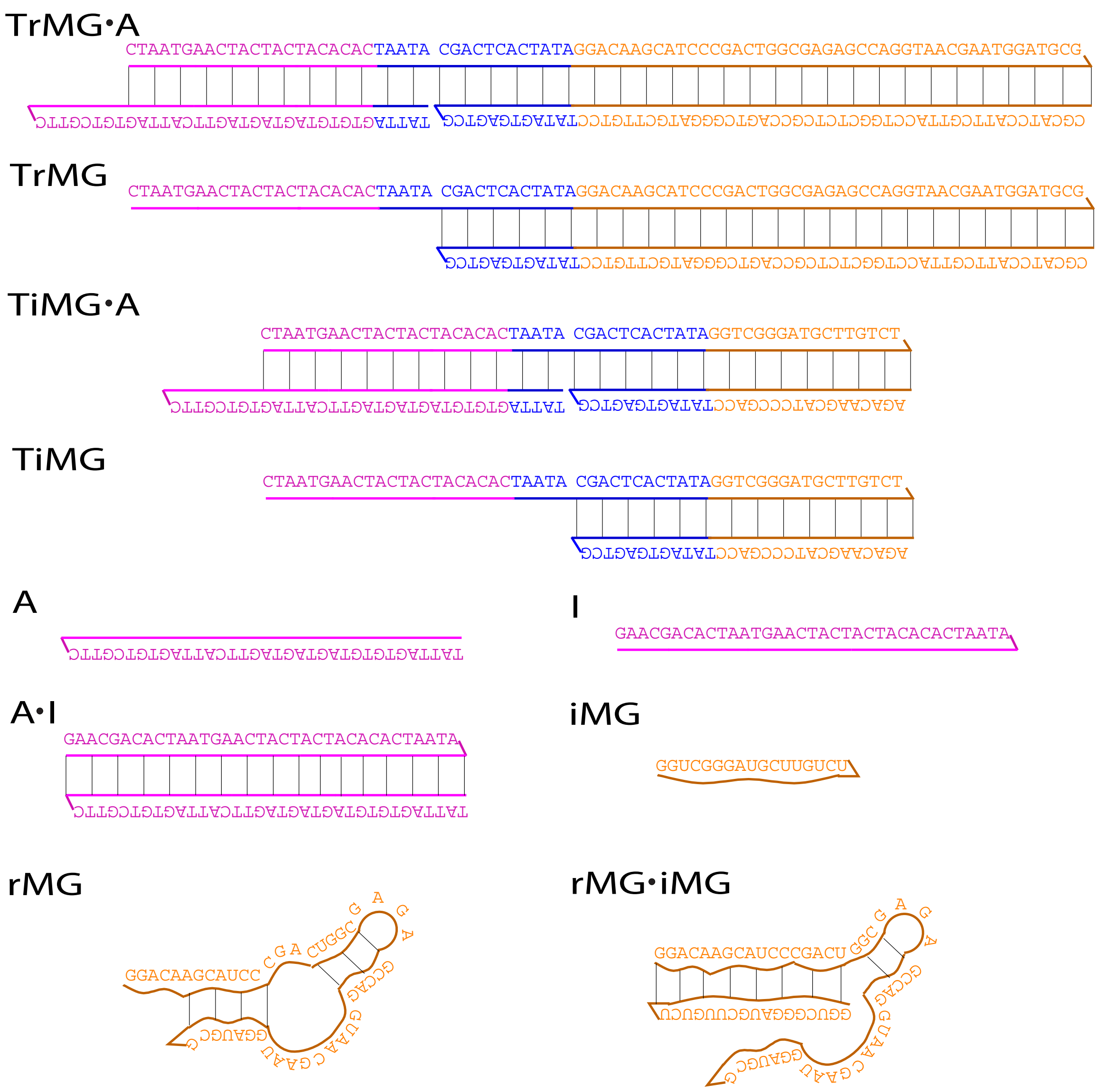

Figure S1: DNA and RNA sequences of single-stranded species and complexes for the synthetic adapter circuit. The sequence domains are color-coded to indicate identical or complementary sequences: magenta indicates the input domain; dark blue indicates the T7 RNAP promoter; orange indicates the output domain. DNA strands are drawn as straight lines; RNA strands are illustrated as squiggly lines. The two DNA strands, TrMG-nt and TrMG-t, comprise the switch template TrMG and similarly TiMG-nt and TiMG-t form TiMG. For the RNA outputs, the predicted secondary structures are shown: iMG does not have a significant secondary structure, whereas rMG contains a central loop for MG dye with a single base of 3′ end exposed. Note that the formation of rMG·iMG complex breaks open the central loop for MG dye and leaves a long domain of 3′ end of rMG exposed while the previously exposed 3′ end of iMG is hidden within the toehold binding domain of rMG.

Table S1: Reaction pathways for the transcriptional circuit

Reaction type	Reaction		
Activation	TiMG + A	$\xrightarrow{k_+}$	TiMG·A
Activation	TrMG + A	$\xrightarrow{k_+}$	TrMG·A
Production	TiMG·A	$\xrightarrow{k_{p1}}$	TiMG·A + iMG
Production	TrMG·A	$\xrightarrow{k_{p2}}$	TrMG·A + rMG
Degradation	iMG	$\xrightarrow{\beta_1}$	ϕ
Degradation	rMG	$\xrightarrow{\beta_2}$	ϕ
Degradation	rMG·iMG	$\xrightarrow{\gamma_1}$	iMG
Degradation	rMG·iMG	$\xrightarrow{\gamma_2}$	rMG
Inhibition	rMG + iMG	$\xrightarrow{k}$	rMG·iMG

Since TrMG and TiMG have identical input domains, the activator A will bind to TrMG and TiMG with the same affinity; the binding reaction will be fast and practically irreversible because of the large gain in thermodynamic energy upon binding. If $[\mathrm{TrMG^{tot}}] + [\mathrm{TiMG^{tot}}] > [\mathrm{A^{tot}}]$, at steady-state all of the activator molecules will be bound to either of the templates: $[\mathrm{A}] \simeq 0$ and $[\mathrm{TrMG \cdot A}] + [\mathrm{TiMG \cdot A}] \simeq [\mathrm{A^{tot}}]$. Thus, the population of A is split between the two templates in proportion with their concentrations after the initial transient,

$$[\mathrm{TiMG \cdot A}] = [\mathrm{A^{tot}}]\frac{[\mathrm{TiMG^{tot}}]}{[\mathrm{TrMG^{tot}}] + [\mathrm{TiMG^{tot}}]}, \quad [\mathrm{TrMG \cdot A}] = [\mathrm{A^{tot}}]\frac{[\mathrm{TrMG^{tot}}]}{[\mathrm{TrMG^{tot}}] + [\mathrm{TiMG^{tot}}]}.$$

Therefore, we can calculate α_1 and α_2 as follows:

$$\alpha_1 = k_{p1}\frac{[\mathrm{TiMG^{tot}}]}{[\mathrm{TrMG^{tot}}] + [\mathrm{TiMG^{tot}}]}, \quad \alpha_2 = k_{p2}\frac{[\mathrm{TrMG^{tot}}]}{[\mathrm{TrMG^{tot}}] + [\mathrm{TiMG^{tot}}]}.$$

The parameters α_1 and α_2 can be continuously tuned by adjusting template concentrations. Due to the binding requirements of RNase R [1], we expect that the degradation of rMG by RNase R would be negligible, i.e. $\beta_2 \simeq 0$ (see Figure S6). Similarly, we expect that the degradation of iMG within rMG·iMG complex by RNase R would be neglibible, i.e. $\gamma_2 \simeq 0$ (see Figure S7 and discussion thereof). Then, the dynamic equations can be rewritten as follows:

$$\begin{aligned} \dot{x} &= \alpha_1 u - \beta_1 x - kxy + \gamma_1 z, \\ \dot{y} &= \alpha_2 u - kxy, \\ \dot{z} &= kxy - \gamma_1 z. \end{aligned} \tag{3}$$

The system has a unique equilibrium point if $u > 0$:

$$\bar{x} = \frac{\alpha_1 u}{\beta_1}, \quad \bar{y} = \frac{\alpha_2 \beta_1}{\alpha_1 k}, \quad \bar{z} = \frac{\alpha_2 u}{\gamma_1}.$$

The steady-state value of output y in this model is independent of input u, showing that the circuit exhibits property of exact adaptation. (When $u = 0$, the steady-states for x and z are determined to be zero, i.e. $\bar{x} = \bar{z} = 0$, but $\bar{y}$ can take any value.)

Michaelis–Menten enzyme kinetics

One of the limitations in the above mathematical model is the assumed first-order enzyme reactions. A more realistic model would use Michaelis–Menten enzyme reactions, where the available enzyme concentrations can be

calculated as follows:

$$\begin{aligned}
[\text{RNAP}] &= \frac{[\text{RNAP}^{\text{tot}}]}{1+\frac{[\text{TrMG}\cdot\text{A}]}{K_{M,TrMGA}}+\frac{[\text{TiMG}\cdot\text{A}]}{K_{M,TiMGA}}+\frac{[\text{TrMG}]}{K_{M,TrMG}}+\frac{[\text{TiMG}]}{K_{M,TiMG}}},\\
&\simeq \frac{[\text{RNAP}^{\text{tot}}]}{1+\frac{[\text{TrMG}\cdot\text{A}]}{K_{M,TrMGA}}+\frac{[\text{TiMG}\cdot\text{A}]}{K_{M,TiMGA}}} \simeq \frac{[\text{RNAP}^{\text{tot}}]}{1+\frac{[\text{A}^{\text{tot}}]}{K_M}},\\
[\text{RNase R}] &= \frac{[\text{RNase R}^{\text{tot}}]}{1+\frac{[\text{rMG}]}{K_{M,R,rMG}}+\frac{[\text{iMG}]}{K_{M,R,iMG}}+\frac{[\text{rMG}\cdot\text{iMG}]}{K_{M,R,rMGiMG}}},\\
&\simeq \frac{[\text{RNase R}^{\text{tot}}]}{1+\frac{[\text{iMG}]}{K_{M,R,iMG}}+\frac{[\text{rMG}\cdot\text{iMG}]}{K_{M,R,rMGiMG}}},
\end{aligned}$$

where we assume high $K_{M,TrMG}$, $K_{M,TiMG}$, and $K_{M,R,rMG}$ (e.g. $K_{M,R,rMG} > 5\mu$M) and similar K_M for the two activated templates (since the sequences for the two templates around the promoter region are identical). The Michaelis constants, the affinity of substrates to the enzymes are calculated as $K_M = \frac{k_{OFF}+k_{cat}}{k_{ON}}$.

For instance, consider the production rate for rMG using TrMG·A as the template for RNAP:

$$\begin{aligned}
\frac{d[\text{rMG}]}{dt} &= k_{cat,rMG}[\text{TrMG}\cdot\text{A}\cdot\text{RNAP}] = k_{cat,rMG}\frac{[\text{TrMG}\cdot\text{A}][\text{RNAP}]}{K_M},\\
&= \frac{k_{cat,rMG}}{K_M}\frac{[\text{RNAP}^{\text{tot}}]}{1+\frac{[\text{A}^{\text{tot}}]}{K_M}}[\text{TrMG}\cdot\text{A}],\\
&= \frac{k_{cat,rMG}}{K_M}\frac{[\text{RNAP}^{\text{tot}}]}{1+\frac{[\text{A}^{\text{tot}}]}{K_M}}\frac{[\text{TrMG}^{\text{tot}}]}{[\text{TrMG}^{\text{tot}}]+[\text{TiMG}^{\text{tot}}]}[\text{A}^{\text{tot}}],
\end{aligned}$$

where [TrMG·A] concentration is calculated as before assuming $[\text{TrMG}^{\text{tot}}]+[\text{TiMG}^{\text{tot}}] > [\text{A}^{\text{tot}}]$. This can be simplified as $\alpha_2[\text{A}^{\text{tot}}]f([\text{A}^{\text{tot}}])$ where α_2 and f are defined as follows:

$$\alpha_2 = \frac{k_{cat,rMG}}{K_M}[\text{RNAP}^{\text{tot}}]\frac{[\text{TrMG}^{\text{tot}}]}{[\text{TrMG}^{\text{tot}}]+[\text{TiMG}^{\text{tot}}]}, \quad f([\text{A}^{\text{tot}}]) = \frac{1}{1+\frac{[\text{A}^{\text{tot}}]}{K_M}}.$$

Note that the value for α_2 is identical to the previous first-order model if we take $k_{p2} = \frac{k_{cat,rMG}}{K_M}[\text{RNAP}^{\text{tot}}]$. Similarly, the production rate for iMG using TiMG·A as the template for RNAP is $\alpha_1[\text{A}^{\text{tot}}]f([\text{A}^{\text{tot}}])$ where f is as defined previous and α_1 is as follows:

$$\alpha_1 = \frac{k_{cat,iMG}}{K_M}[\text{RNAP}^{\text{tot}}]\frac{[\text{TiMG}^{\text{tot}}]}{[\text{TrMG}^{\text{tot}}]+[\text{TiMG}^{\text{tot}}]}.$$

The degradation rates for iMG and rMG·iMG can be analogously transformed to the corresponding Michaelis–Menten enzyme reactions using an appropriate enzyme saturation term for RNase R:

$$g([\text{iMG}],[\text{rMG}\cdot\text{iMG}]) = \frac{1}{1+\frac{[\text{iMG}]}{K_{M,R,iMG}}+\frac{[\text{rMG}\cdot\text{iMG}]}{K_{M,R,rMGiMG}}}.$$

Let $u = [\text{A}^{\text{tot}}]$, $x = [\text{iMG}]$, $y = [\text{rMG}]$, and $z = [\text{rMG}\cdot\text{iMG}]$. Then the dynamics for the system can be rewritten as follows similar to eqs. (3):

$$\begin{aligned}
\dot{x} &= \alpha_1 u f(u) - \beta_1 x g(x,z) - kxy + \gamma_1 z g(x,z),\\
\dot{y} &= \alpha_2 u f(u) - kxy,\\
\dot{z} &= kxy - \gamma_1 z g(x,z).
\end{aligned} \tag{4}$$

Note that the derivations above used the standard Michaelis–Menten assumption that substrates are in excess of enzymes – enzymes used in excess of substrates help reduce the saturation of enzymes. For the rest of the paper,

we will assume that the enzymes are in excess and/or the substrate concentrations are suitably lower than their respective Michaelis constants such that $f(u) \approx 1$ and $g(x,z) \approx 1$ – i.e. the dynamics of the system essentially follow eqs. (3).

Non-dimensional equations

We rescale variables with respect to their steady-state values,

$$\hat{u} = \frac{u}{u_0}, \;\; \hat{x} = \frac{x}{\bar{x}}, \;\; \hat{y} = \frac{y}{\bar{y}}, \;\; \hat{z} = \frac{z}{\bar{z}},$$

such that all normalized variables have values of 1 at steady-state. The timescale is normalized by the degradation rate of species x: $\tau = \beta_1 t$. For example, equation for z is rewritten as follows:

$$\begin{aligned} \frac{d}{dt} z &= kxy - \gamma_1 z, \\ \frac{1}{\beta_1}\frac{d}{dt}\hat{z}\bar{z} &= \frac{k}{\beta_1}\hat{x}\bar{x}\hat{y}\bar{y} - \frac{\gamma_1}{\beta_1}\hat{z}\bar{z}, \\ \frac{d}{d\tau}\hat{z} &= \frac{k}{\beta_1}\frac{\bar{x}\bar{y}}{\bar{z}}\hat{x}\hat{y} - \frac{\gamma_1}{\beta_1}\hat{z}, \\ \frac{d}{d\tau}\hat{z} &= \frac{\gamma_1}{\beta_1}\left(\hat{x}\hat{y} - \hat{z}\right). \end{aligned}$$

Upon change of variables to non-dimensional ones as above (we dropped the $\hat{}$'s with a slight abuse of notations), eqs. (3) can be expressed as follows:

$$\begin{aligned} \dot{x} &= u - x - p_1(xy - z), \\ \dot{y} &= p_2(u - xy), \\ \dot{z} &= p_3(xy - z), \end{aligned} \tag{5}$$

where $p_1 = \frac{\alpha_2}{\alpha_1}$, $p_2 = \frac{1}{\beta_1}\frac{k\alpha_1 u_0}{\beta_1}$ and $p_3 = \frac{\gamma_1}{\beta_1}$.

Integral feedback

It is known that any system that perfectly adapts to a step input, including an incoherent feedforward loop can be transformed, possibly with a nonlinear state transformation, into an integral feedback form [2, 3]. Accordingly, the set of eqs. (5) can be represented in the integral feedback form through the following choice of the coordinate transformation $(x, y, z) \to (I, y, z)$ where $I = x - \frac{1}{p_2}y + \frac{p_1}{p_3}z$. In particular, the dynamics of I satisfy the integral feedback form as follows:

$$\dot{I} = x(y - 1).$$

3. Characterization of Elementary Reactions

We observed that the bleaching of free MG dye in the transcription reaction buffer is not negligible for long experiments. The bleaching of MG dye is reported to follow a pseudo-first order kinetics [4]; thus, an exponential curve was fit to the fluorescence data with a decay rate of 0.04/hr (Figure S2A). However, the presence of MG aptamer is reported to suppress the bleaching of MG dye [5]. Since the MG dye in the system is in great excess as compared to the MG aptamer ([MG dye]=25 μM, [rMG]$<$ 1 μM for all experiments), we applied the same correction for bleaching of MG dye to all fluorescence trajectories. The fluorescence increase upon addition of 1 μM of purified rMG was determined to be 500,000 counts in the presence of MG dye; this was used to convert the fluorescence reading to aptamer concentrations after background correction.

First, the transcription reactions for the two templates, TrMG and TiMG, are characterized through fluorescence measurements on spectrofluorometer. The template TrMG is formed by annealing the two DNA strands TrMG-nt and TrMG-t: the resulting template contains 5 bases of promoter region missing on the template side, and thus transcribes poorly [6] (cf. Figure S1). When the input A that complements the missing promoter region is added, the template TrMG·A can be transcribed well. The transcription of rMG is monitored in real time in the presence of 25 μM MG dye (Figure S3). Except for the initial burst phase, which showed potential saturation for high template concentration

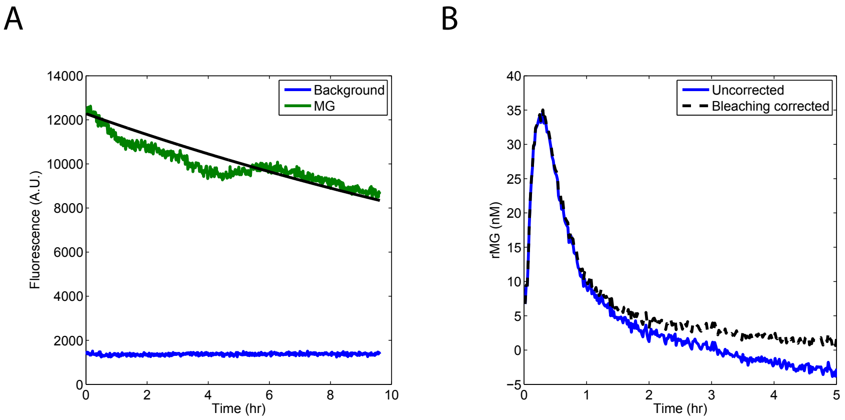

Figure S2: Determination of bleaching rates for MG dye and baseline correction. (**A**) To determine the bleaching rate of free MG dye, 25 μM MG dye was included for MG trajectories (green curve). An exponential decay curve ($\lambda = 0.04$/hr) was fit to the data (black curve). The background fluorescence did not significantly change when no MG dye was included in the reaction buffer (blue curve). The measurement conditions were identical to all the other spectrofluorometer measurements (excitation wavelength: 630 nm, emission wavelength: 655 nm, slit widths: 5 nm, integration time: 0.5 s, measurement interval: 1 min, temperature: 33°C). (**B**) Using the initial measurement of baseline fluorescence as 0 nM rMG concentration without further baseline correction, the converted rMG concentration apparently became negative because the experimental trajectory went below the initial baseline fluorescence; the predicted steady-state of rMG was 0 nM in this case (cf. blue curve in Figure S9B). The bleaching curve for free MG dye was used to correct for the baseline: the corrected curve does not go below zero for converted rMG concentrations (black dashed line).

([TrMG^{tot}]), the steady-state transcription rate (k_{p2}[TrMG·A]) increased as the activator concentration increased; in this case, [TrMG·A]$\simeq$[A^{tot}] because [TrMG^{tot}]>[A^{tot}]. The fluorescence signal increase due to rMG transcription appeared approximately a minute after the addition of A, indicating that the binding reaction between TrMG and A is fast (large k_+). The normalized slopes for rMG transcription (k_{p2}[TrMG·A]/[A^{tot}]$\simeq k_{p2}$) are approximately 0.5/min.

However, the transcription rate was apparently not constant for the initial part of transcription reaction reminiscent of 'burst phase' in enzyme reaction [7]. (However, the timescale for typical burst phase is short for transcription studies with T7 RNAP [8].) It has been reported that strong secondary structure of RNA transcript reduces the transcription rate by T7 RNAP [9]. One hypothesis we can explore is that the change of transcription rate is dependent on the conformation change of DNA templates: the annealed fully duplex template serves as an efficient template for RNAP at the beginning of transcription; however, because the rMG transcript has a strong secondary structure, the duplex template may take a cruciform or hairpin structure at some rate upon transcription by RNAP which in turn reduces the efficiency of transcription. Thus, we use the following equation to fit the transcription rate for rMG:

$$k_{p2,burst} = k_{p2}(1 + Be^{-t/\tau_b}),$$

where B is the magnitude of increased transcription efficiency during the initial burst phase and τ_b is the time constant for duration of burst phase. The fitting result showed $k_{p2} = 0.006$/s, $B = 3.8$ and $\tau_b = 2500$s for activator concentrations ranging from 20 to 80 nM (Figure S3A). The highest activator concentration resulted in less than expected burst phase possibly due to saturation of enzyme dynamics. We note that the fit is phenomenological; a mechanistic understanding would require further characterization.

Similarly, the template TiMG that codes for the inhibitor of rMG, iMG, contains the same input domain architecture as TrMG; therefore, the same input A can activate the transcription of iMG. The transcription of iMG can be monitored real time using fluorescence change; as iMG is transcribed, iMG binds to rMG and disrupts the binding pocket of rMG for MG dye (cf. Figure S1), which decreases the fluorescence (Figure S4). For the case of iMG production, the 'burst phase' was not apparently observed possibly because the iMG transcript does not have a significant secondary structure. The steady-state transcription rate (k_{p1}[TiMG·A]) increased as the activator concentration increased; again [TiMG·A]$\simeq$[A^{tot}] because [TiMG^{tot}]>[A^{tot}]. The normalized slopes for iMG transcription

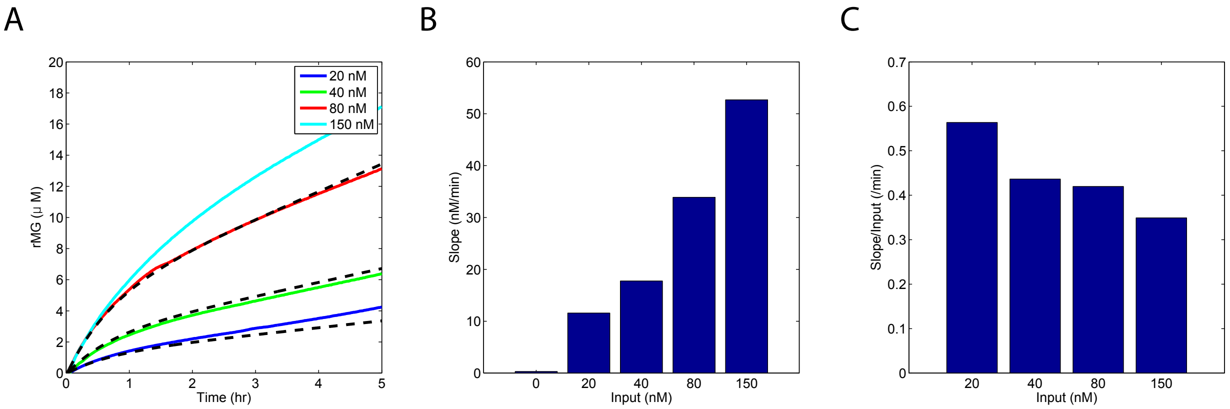

Figure S3: Determination of transcription rates for rMG (α_2). (**A**) As rMG is being transcribed, the fluorescence signal from rMG increased over time. Fluorescence data were converted to rMG concentrations using offline measurements of fluorescence signals from purified rMG as standards. The experimental conditions were as follows: [TrMG^{tot}] = 50 nM for low inputs (20 and 40 nM), [TrMG^{tot}] = 150 nM for high inputs (80 and 150 nM), and [RNAP] = 171 nM. The fit results using burst phase model for activator concentrations ranging from 20 to 80 nM are shown as black dashed lines. This graph is shown in Figure 3A of the main text. (**B**) The steady-state slopes of curves from (A) were determined as follows: the average slope were calculated between 2 hr and 3 hr points for all input concentrations to exclude the initial burst phase. The average slope for the initial 30 min prior to the addition of inputs was taken as the slope for no input. (**C**) The steady-state slopes in (B) were normalized by the amount of activator, i.e., the amount of active template TrMG·A. The normalized slopes (k_{p2}) were approximately 0.5/min for activator concentrations ranging from 20 to 150 nM.

(k_{p1}[TiMG·A]/[A^{tot}]$\simeq k_{p1}$) are approximately 1/min.

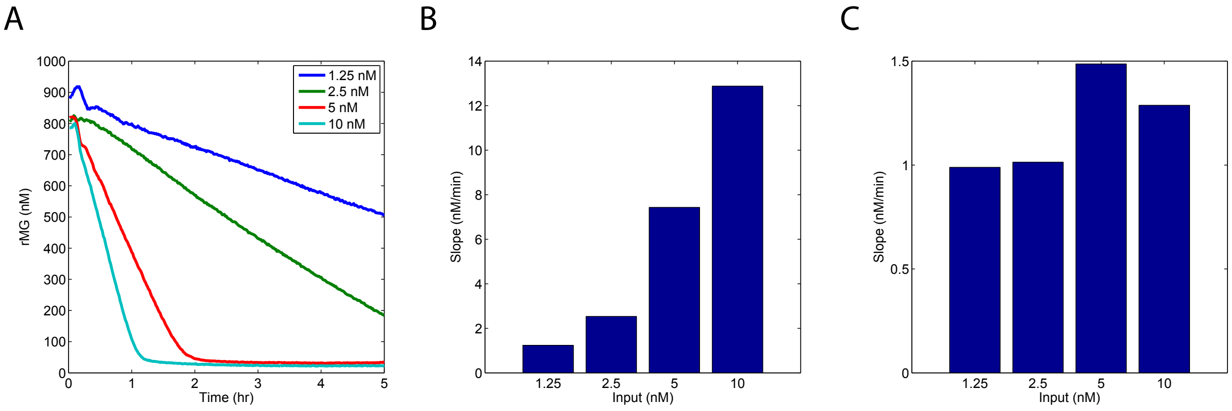

Figure S4: Determination of transcription rates for iMG (α_1). (**A**) The transcription of iMG induced the reduction of rMG fluorescence over time as rMG·iMG complexes are formed. Fluorescence data were converted to rMG concentrations using offline measurements of fluorescence signals from purified rMG as standards. The experimental conditions were as follows: initial [rMG] = 1 μM, [TiMG] = 50 nM, and [RNAP] = 171 nM. (**B**) The slopes of curves from (A) were determined as follows: the average slope were calculated between 2 hr and 3 hr points for 1.25 nM and 2.5 nM inputs, and the slope between 30 min and 1 hr points for 5 nM and 10 nM inputs. (**C**) The slopes in (B) were normalized by the amount of activators, i.e., the amount of active template TiMG·A. The normalized slopes (k_{p1}) were approximately 1/min for activator concentrations ranging from 1.25 to 10 nM.

Next, the binding reaction for the two RNA signals, rMG and iMG, is characterized through fluorescence measurements. Initially, 10 nM rMG and 25 μM MG dye are incubated in the cuvette to establish baseline fluorescence.

Then, 10 nM iMG is added to the cuvette and the decrease of fluorescence is monitored as rMG binds to iMG to form rMG·iMG complex. (Using only 10 nM rMG made the fluorescence signal a bit noisy due to overall low signal level; however, using high concentrations of rMG and iMG would make the binding reaction proceed too quickly to yield suitable number of data points.) Because the two single-stranded species have the same concentration, the following equations can be used to find k:

$$\frac{d}{dt}[\mathrm{rMG}\cdot\mathrm{iMG}] = k[\mathrm{rMG}][\mathrm{iMG}] = k[\mathrm{rMG}]^2,$$

and thus,

$$\frac{d}{dt}[\mathrm{rMG}] = -k[\mathrm{rMG}][\mathrm{iMG}] = -k[\mathrm{rMG}]^2.$$

By integrating the above equation, we find

$$\frac{1}{[\mathrm{rMG}_0]} - \frac{1}{[\mathrm{rMG}]} = -kt,$$

where $[\mathrm{rMG}_0]$ is the initial concentration of rMG. Figure S5C illustrates the linear fit using the above relations for duplicate experiments. The fitted binding rate constant k is 4.2×10^4/M/s, consistent with typical toehold-mediated branch migration reactions [10, 11]. The resulting fit can be used to plot fit curves as shown in Figure S5B.

$$[\mathrm{rMG}] = \frac{1}{kt + 1/[\mathrm{rMG}_0]}$$

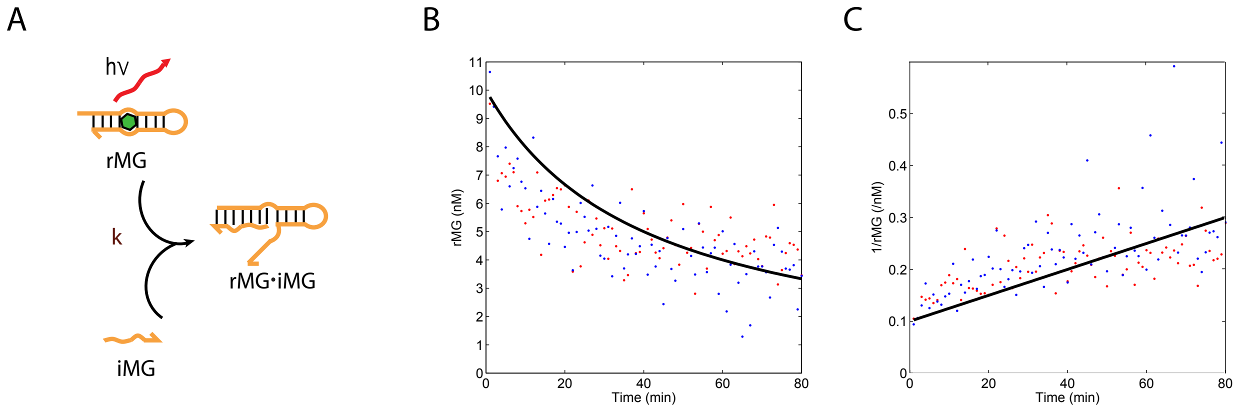

Figure S5: Determination of binding rates for rMG and iMG (k). (**A**) Schematic of the hybridization reaction of rMG and iMG. (**B**) The hybridization reaction of rMG and iMG was characterized by adding 10 nM of iMG to 10 nM of rMG. The data points for duplicate reactions are plotted as red and blue dots. The black line is the fit from (C): $[\mathrm{rMG}] = 1/(kt + 0.1)$. This graph is shown in Figure 3B of the main text. (**C**) To facilitate fitting for the second-order hybridization reactions, the inverse of rMG concentrations (1/[rMG]) are plotted. The black line is the result of linear fit: $1/[\mathrm{rMG}] = kt + 1/[\mathrm{rMG}_0] = kt + 0.1$ with $k = 0.0025$/nM/min= 4.2×10^4/M/s.

Finally, we characterize the degradation of RNA signals catalyzed by RNase R. Due to the binding requirements of RNase R, RNA sequences with less than 4 nt of single-strand regions exposed at the 3′ end would be degraded poorly by RNase R [1]. To minimize the degradation rate of rMG by RNase R (β_2), the initial design of rMG contained no available single-stranded region at its 3′ end. However, we observed that having the 3′ end perfectly paired to the 5′ domain of rMG caused non-specific extension of RNA transcripts for rMG [12]. Therefore, we placed a single unpaired base at the 3′ end of rMG (cf. Figure S1). (We typically place three unpaired bases at the 3′ end of RNA signals used in transcriptional circuits [13, 6].) The degradation rate of rMG by RNase R was monitored by fluorescence and gel. As shown in Figure S6, the degradation of rMG alone by RNase R was negligible ($\beta_2 \simeq 0$).

On the other hand, the other RNA signal iMG is designed to have no significant secondary structure, presumably providing a long single-stranded region at its 3′ end (cf. Figure S1). The degradation of iMG by RNase R was fast:

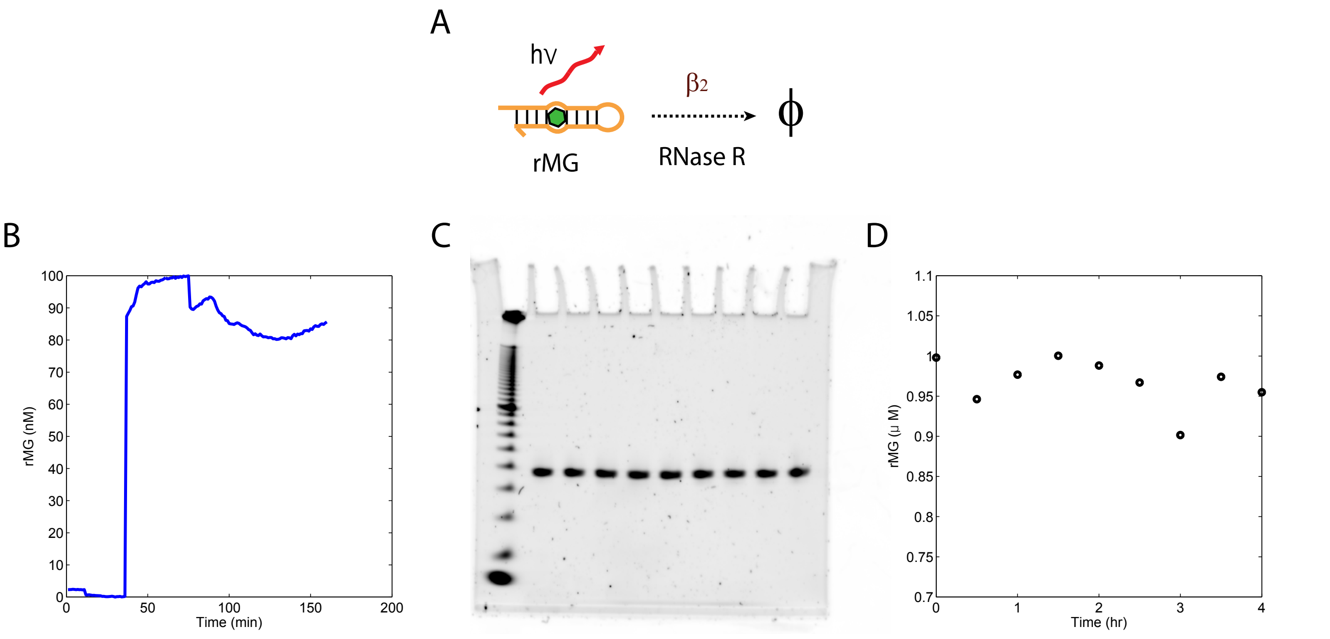

Figure S6: Determination of degradation rates for rMG (β_2). (**A**) Schematic of the degradation reaction of rMG. (**B**) The degradation reaction of rMG was characterized by adding 75 nM of RNase R to 100 nM of rMG using Spectrofluorometer. (**C**) The degradation reaction of rMG was characterized by adding 75 nM of RNase R to 1 μM of rMG using denaturing gel. Left most lane contains 10 bp ladder; other lanes contain samples incubated for 30 min increments from left to right. (**D**) Quantitation of rMG bands in the gel shown in (C).

1 μM of iMG was completely degraded by 75 nM RNase R in less than 30 min as determined by gel (data not shown). The gel analysis did not lead to quantitative estimate of β_1, but it is safe to conclude that $\beta_1 \gg \beta_2$.

The remaining question is the degradation pathway of rMG·iMG complex. Figure S7A illustrates the potential degradation pathways for the rMG·iMG complex: either iMG or rMG within the complex can be processed first by RNase R and the resulting rMG or iMG will be further degraded by RNase R (or participate in a binding reaction). Gel analysis of preformed 1 μM rMG·iMG complex incubated with 75 nM RNase R showed that both rMG and iMG were almost completely degraded within 30 min. Because stoichiometric amount of rMG and iMG were used for gel analysis, if iMG were to be degraded first ($\gamma_2 > \gamma_1$), rMG would be left over which cannot be easily degraded ($\beta_2 \simeq 0$). Thus, we conclude that the degradation of iMG within rMG·iMG complex is very slow ($\gamma_2 \simeq 0$). To obtain estimates for parameter values, we performed a spectrofluorometer experiment where a substoichiometric amount of iMG was used with respect to rMG. Specifically, 100 nM of iMG was allowed to bind to 1 μM of rMG in the presence of MG dye. Upon addition of RNase R, the fluorescence signal from rMG decreased quickly to about 750 nM rMG. This suggests that the iMG molecule released upon degradation of rMG within the rMG·iMG complex can bind to free rMG molecules such that it catalyzes the degradation of multiple rMG molecules until the iMG within the system is exhausted. If the iMG within the rMG·iMG complex were to be degraded first ($\gamma_2 > \gamma_1$), the resulting rMG molecule will increase fluorescence. This was not observed in the spectrofluorometer experiment. Thus, we conclude that the design strategy that limits exposure of $3'$ end of iMG upon rMG·iMG complex formation while releasing the hidden $3'$ end of rMG results in fast degradation of rMG within rMG·iMG complex ($\gamma_1 \gg 0$ and $\gamma_2 \simeq 0$). Unfortunately, it is difficult to separately characterize β_1 and γ_1 since both rMG·iMG complex and iMG molecule have similarly low fluorescence. Recognizing that the addition of RNase R may affect the hybridization reaction between the two RNA species possibly due to its interaction at the $3'$ ends of RNA molecules, we optimized the parameters for k, β_1, and γ_1 simultaneously. The fitting result showed $k = 3.5 \times 10^4$/M/s, $\beta_1 = 0.0176$/s, and $\gamma_1 = 0.0095$/s (Figure S7).

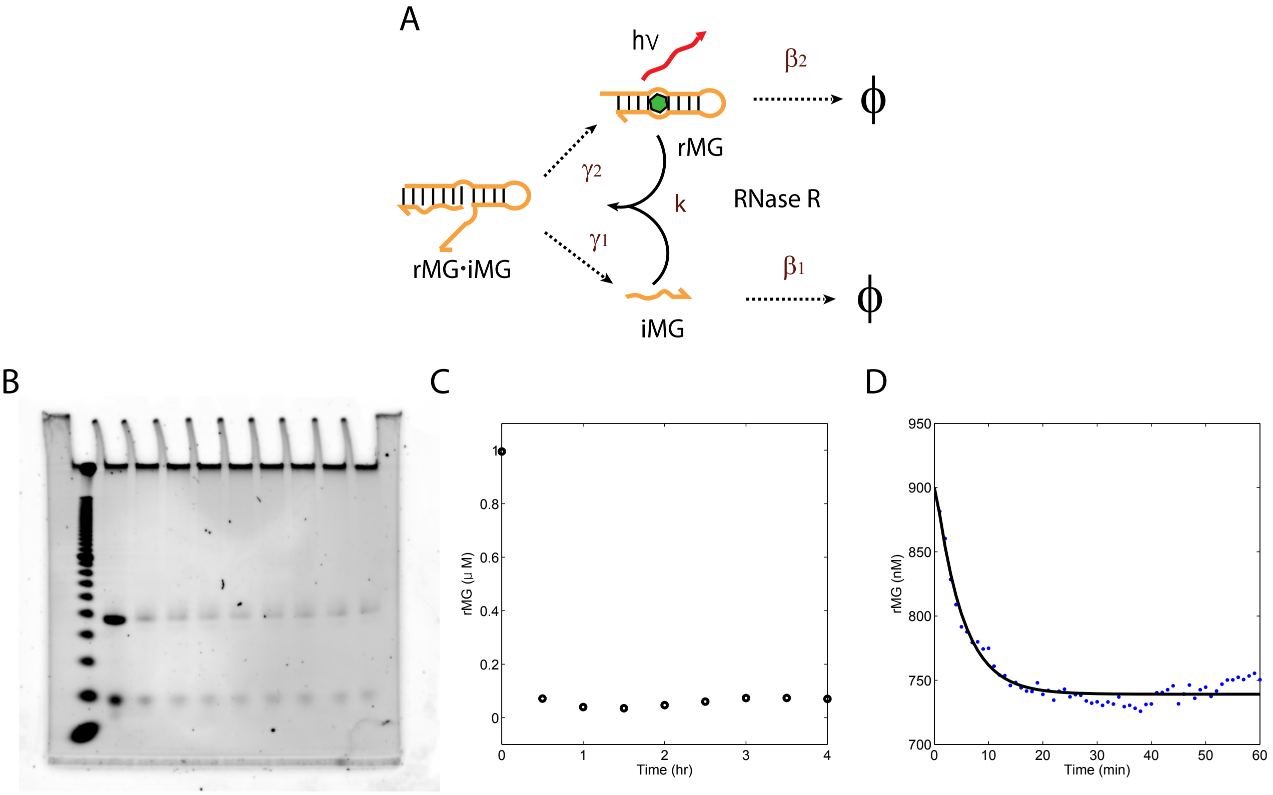

Figure S7: Determination of degradation rates for rMG·iMG complex (β_1 and γ_1). (**A**) Schematic of the degradation reaction of rMG·iMG complex. (**B**) The degradation reaction of rMG·iMG complex was characterized by adding 75 nM of RNase R to 1 μM of rMG·iMG complex using denaturing gel. Here, the degradation is complete because stoichiometric amounts of rMG and iMG were used. Left most lane contains 10 bp ladder; other lanes contain samples incubated for 30 min increments from left to right. (**C**) Quantitation of rMG bands in the gel shown in (B). (**D**) The degradation reaction of rMG·iMG complex was characterized by adding 75 nM of RNase R to 100 nM of iMG and 1 μM rMG using Spectrofluorometer. The degradation of rMG stopped in the middle presumably because all the iMG molecules were exhausted. The timecourse immediately after the addition of RNase R is shown as blue dots. The black line is the fit using $k = 3.5 \times 10^4$/M/s, $\beta_1 = 0.0176$/s and $\gamma_1 = 0.0095$/s. This graph is shown in Figure 3C of the main text.

Table S2: Kinetic parameters from analyzing elementary reactions and the whole circuit.

parameters	elementary reactions	circuit	lower bound	upper bound
k_{p1} (/s)	0.016	0.027	0.003	0.03
k_{p2} (/s)	0.006	0.015	0.003	0.03
β_1 (/s)	0.0176	0.0018	0.0003	0.03
γ_1 (/s)	0.0095	0.0011	0.0003	0.03
k (/M/s)	3.5×10^4	1.5×10^4	10^3	10^5
k_+ (/M/s)	–	10^5	10^3	10^5
B	3.8	1.7	1	10
τ_b (s)	2.5×10^3	3.2×10^3	10^3	10^4

*k_{p1}, k_{p2}, B, and τ_b are for the experiments with 171 nM RNAP;
β_1 and γ_1 are for the experiments with 75 nM RNase R.

References

[1] Vincent, H. A. and Deutscher, M. P. (2006) Substrate recognition and catalysis by the exoribonuclease RNase R. *J Biol Chem*, **281**, 29769–29775.

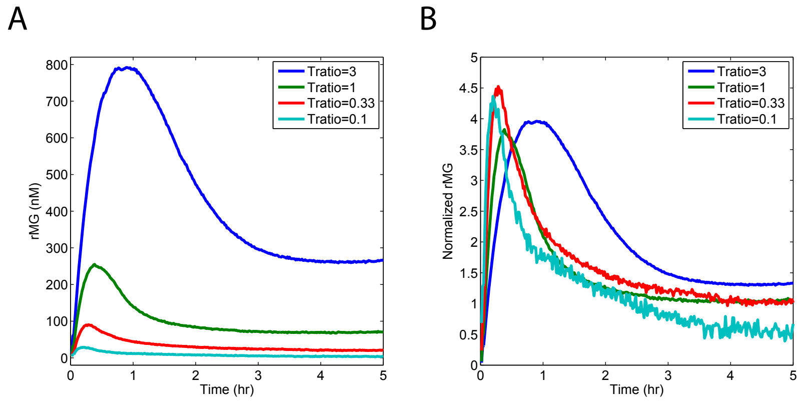

Figure S8: Circuit responses for different choice of template ratios. (**A**) The template ratio ($[\text{TrMG}^{\text{tot}}]/[\text{TiMG}^{\text{tot}}]$) was varied while keeping the total concentration of templates fixed ($[\text{TrMG}^{\text{tot}}] + [\text{TiMG}^{\text{tot}}] = 200$ nM). (**B**) The circuit responses are normalized to the expected steady-state values. For the fluorescence trace with the lowest rMG signal, the bleaching of MG fluorescence was significant. The presence of MG aptamer is shown to slow down the bleaching of MG fluorescence [5], helping explain more stable fluorescence signals in other traces.

[2] Shoval, O., Goentoro, L., Hart, Y., Mayo, A., Sontag, E., and Alon, U. (2010) Fold-change detection and scalar symmetry of sensory input fields. *Proc Natl Acad Sci USA*, **107**, 15995–16000.

[3] Sontag, E. D. (2010) Remarks on feedforward circuits, adaptation, and pulse memory. *IET Syst Biol*, **4**, 39–51.

[4] Gandhi, J., Dangi, R., Sharma, J. C., Verma, N., and Bhardwaj, S. (2010) Photocatalytic bleaching of malachite green and brilliant green dyes using zns-cds as semiconductor: A comparative study. *Der Chemica Sinica*, **1**, 77–83.

[5] Wang, T. (2008) *Function and dynamics of aptamers: A case study on the malachite green aptamer*. Ph.D. thesis, Iowa State University, Department of Molecular, Cellular and Developmental Biology.

[6] Kim, J., White, K. S., and Winfree, E. (2006) Construction of an *in vitro* bistable circuit from synthetic transcriptional switches. *Mol. Syst. Biol.*, **2**, 68.

[7] Jia, Y. and Patel, S. S. (1997) Kinetic mechanism of transcription initiation by bacteriophage T7 RNA polymerase. *Biochemistry*, **36**, 4223–4232.

[8] Kuzmine, I. and Martin, C. T. (2001) Pre-steady-state kinetics of initiation of transcription by T7 RNA polymerase: A new kinetic model. *J. Mol. Biol.*, **305**, 559–566.

[9] Nagatoishi, S., Ono, R., and Sugimoto, N. (2012) The yields of transcripts for a RNA polymerase regulated by hairpin structures in nascent RNAs. *Chem Commun*, **48**, 5121–5123.

[10] Yurke, B. and Mills, A. P., Jr. (2003) Using DNA to power nanostructures. *Genetic Programming and Evolvable Machines*, **4**, 111–122.

[11] Zhang, D. Y. and Winfree, E. (2009) Control of DNA strand displacement kinetics using toehold exchange. *J Am Chem Soc*, **131**, 17303–17314.

[12] Triana-Alonso, F. J., Dabrowski, M., Wadzack, J., and Nierhaus, K. H. (1995) Self-coded 3′-extension of run-off transcripts produces abberant products during *in vitro* transcription with T7 RNA polymerase. *J. Biol. Chem.*, **270**, 6298–6307.

[13] Kim, J. and Winfree, E. (2011) Synthetic *in vitro* transcriptional oscillators. *Mol. Syst. Biol.*, **7**, 465.

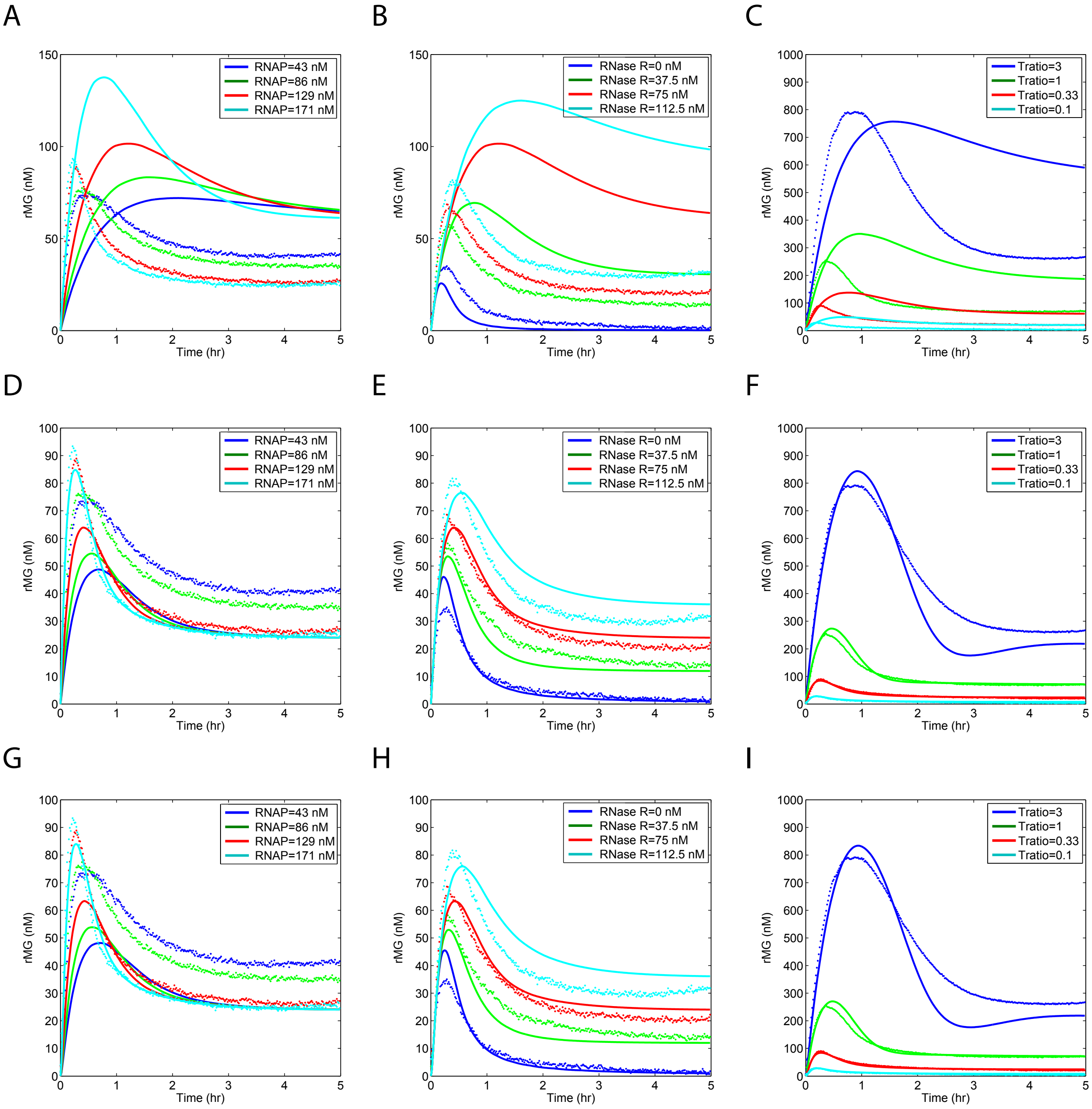

Figure S9: Experimental and simulation results for circuit responses for different concentrations of enzymes and template ratios. (**A–C**) The simple model (eqs. 3) was used for simulation with the 'default' parameters from elementary reactions listed in Table S2. For the simple model, the binding of input A to switch templates are instantaneous and hence are not modeled as separate states. The simulation results qualitatively captured the features of experimental results for RNAP variation (A), RNase R variation (B), and template ratio variation (C). (**D–F**) The simple model (eqs. 3) was used with optimized parameters listed in Table S2. These graphs are shown in Figure 4 of the main text. (**G–I**) The mathematical model for the transcriptional circuit (eqs. 1) was used for simulation where the input A and switch templates are modeled as separate species. The optimized k_+ was at the maximum allowed value (Table S2), and consequently, there were little differences between the simulation results of these two models. The experimental data are plotted as dots; the simulation results are plotted as lines.

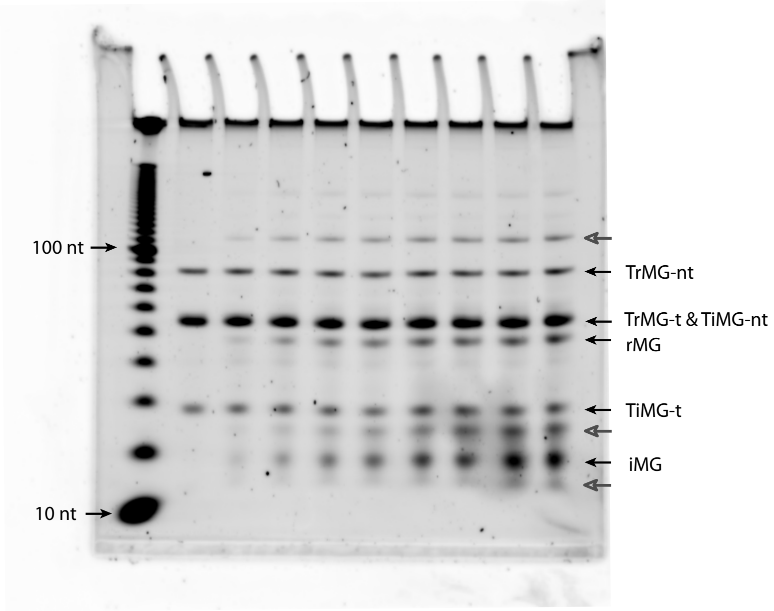

Figure S10: Gel analysis of the transcription reaction. The transcription reaction for rMG and iMG was performed by adding 86 nM of RNAP to 50 nM of TrMG, 150 nM of TiMG, and 10 nM of A. The transcription products were analyzed using denaturing gel. Left most lane contains 10 bp ladder with 10 nt and 100 nt marked; other lanes contain samples incubated for 30 min increments from left to right. The DNA species and RNA transcripts were marked by black arrows with corresponding names: TrMG-t and TiMG-nt differ by a single nt in length and were not resolved separately; A was not clearly identified due to its low concentration. Major bands for unidentified products – possible truncation and non-specific extension products – were marked by grey arrows.

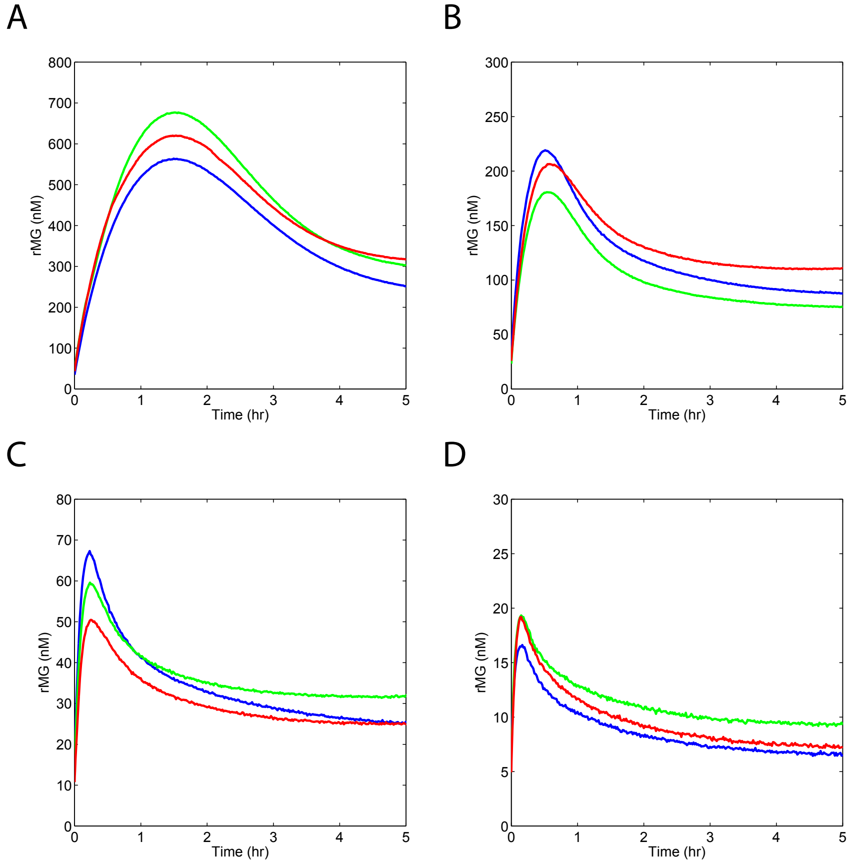

Figure S11: Experimental results for circuit responses using a different batch of enzymes (batch #2). The nominal enzyme concentrations are the same as those in Figure 4C of the main text. (**A–D**) The template ratio ($[\text{TrMG}^{\text{tot}}]/[\text{TiMG}^{\text{tot}}]$) was varied while keeping the total concentration of templates fixed ($[\text{TrMG}^{\text{tot}}] + [\text{TiMG}^{\text{tot}}] = 200$ nM): The template ratios were (A) 3, (B) 1, (C) 0.33, and (D) 0.1. The steady-states reached were proportional to template ratios as expected. The standard deviation for the three trajectories averaged over 5 hr timecourse were (A) 37.2 nM, (B) 16.2 nM, (C) 3.61 nM, and (D) 1.32 nM. The average standard deviations ranged from 12 to 16% of the final steady-state in each experiment, indicating that the adaptation behavior is reproducible.

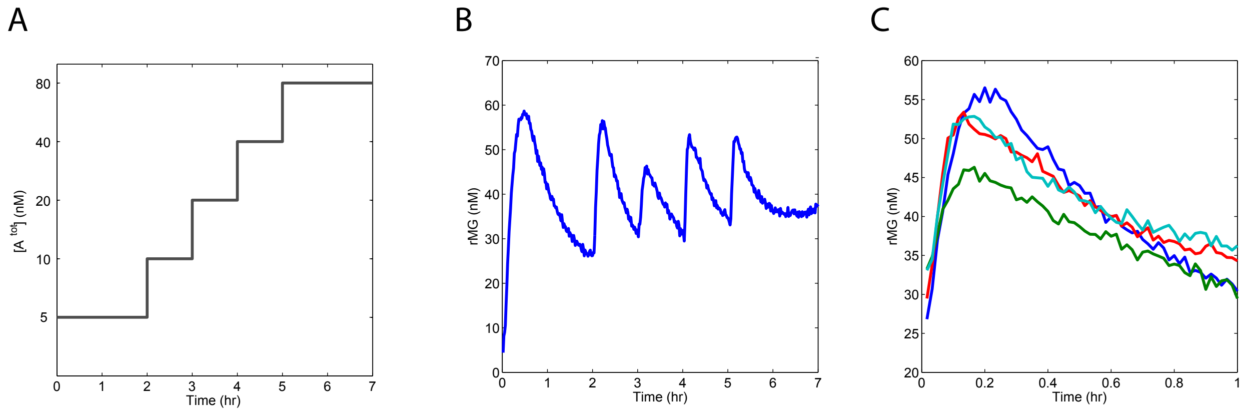

Figure S12: Circuit response follows fold-change of inputs. (**A**) After the initial transient was settled, multiple input perturbations were induced every hour; the total concentration of inputs were increased by 2-fold every time: $[\mathrm{A}^{\mathrm{tot}}] = 5$ nM at time 0, 10 nM at 2 hrs, 20 nM at 3 hrs, 40 nM at 4 hrs, 80 nM at 5 hrs. The template concentrations were $[\mathrm{TrMG}^{\mathrm{tot}}] = 50$ nM and $[\mathrm{TiMG}^{\mathrm{tot}}] = 150$ nM. (**B**) The circuit shows similar level of response after every 2-fold changes of input indicating an approximate fold-change detection. (**C**) The segments of timecourse in (B) are rearranged with respect to time after input perturbations. The second response (green) was smaller than others possibly due to an incomplete mixing of inputs.

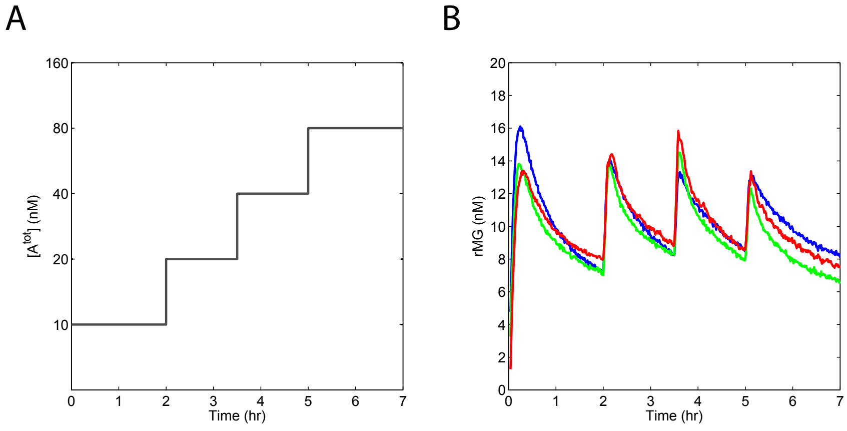

Figure S13: Circuit response follows fold-change of inputs for enzyme batch #2. (**A**) After the initial transient was settled, multiple input perturbations were induced; the total concentration of inputs were increased by 2-fold every time: $[\mathrm{A}^{\mathrm{tot}}] = 10$ nM at time 0, 20 nM at 2 hrs, 40 nM at 3.5 hrs, 80 nM at 5 hrs. The template concentrations were $[\mathrm{TrMG}^{\mathrm{tot}}] = 18$ nM and $[\mathrm{TiMG}^{\mathrm{tot}}] = 180$ nM. (**B**) The circuit shows similar level of response after every 2-fold changes of input indicating an approximate fold-change detection. The standard deviation for the three trajectories averaged over 7 hr timecourse was 0.66 nM, indicating that the fold-change detection is reproducible.
